# Achieving consensus in multilateral international negotiations: The case study of the 2015 Paris Agreement on climate change

**DOI:** 10.1126/sciadv.abg8068

**Published:** 2021-12-15

**Authors:** Carmela Bernardo, Lingfei Wang, Francesco Vasca, Yiguang Hong, Guodong Shi, Claudio Altafini

**Affiliations:** 1 Group for Research on Automatic Control Engineering, Department of Engineering, University of Sannio, 82100, Benevento, Italy.; 2 Key Laboratory of System and Control, Academy of Mathematics and System Science, University of Chinese Academy of Sciences, Beijing 100190, China.; 3 Australian Center for Field Robotics, School of Aerospace, Mechanical and Mechatronic Engineering, The University of Sydney, NSW 2008, Sydney.; 4 Division of Automatic Control, Department of Electrical Engineering, Linköping University, SE-58183 Linköping, Sweden.

## Abstract

The purpose of this paper is to propose a dynamical model describing the achievement of the 2015 Paris Agreement on climate change. To represent the complex, decade-long, multiparty negotiation process that led to the accord, we use a two time scale dynamical model. The short time scale corresponds to the discussion process occurring at each meeting and is represented as a Friedkin-Johnsen model, a dynamical multiparty model in which the parties show stubbornness, i.e., tend to defend their positions during the discussion. The long time scale behavior is determined by concatenating multiple Friedkin-Johnsen models (one for each meeting). The proposed model, tuned on real data extracted from the Paris Agreement meetings, achieves consensus on a time horizon similar to that of the real negotiations. Remarkably, the model is also able to identify a series of parties that exerted a key leadership role in the Paris Agreement negotiation process.

## Teaser

A dynamical model describes the negotiation process that led to the 2015 Paris Agreement on climate change.

## INTRODUCTION

The 2015 Paris Agreement on climate change is an accord ratified by 196 “parties” [195 countries and the European Union (EU)] under the aegis of the United Nations Framework Convention on Climate Change (UNFCCC). Its aim is to coordinate the international efforts to keep the effects of global warming to below 2^∘^C relative to the preindustrial level and to pursue efforts to limit them further to 1.5^∘^C.

**Fig. 1.**
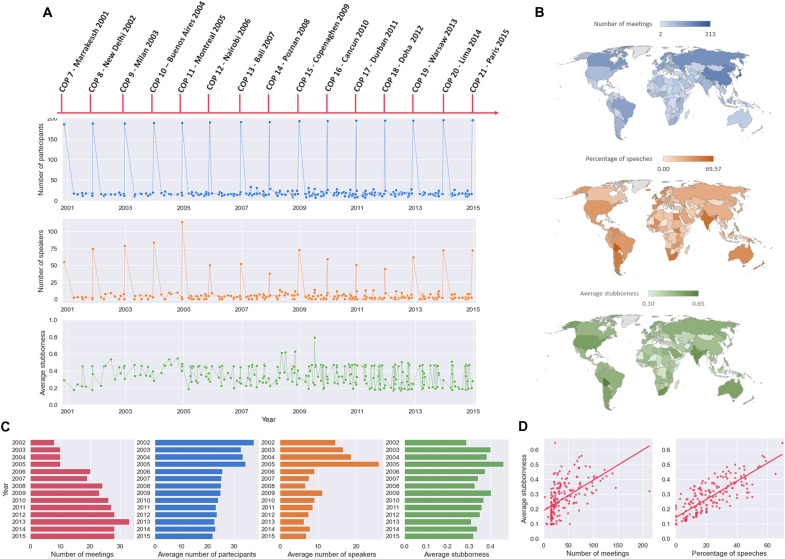
Paris Agreement dataset. (**A**) For the years 2001 to 2015, the number of participants, the number of speakers, and the average stubbornness of each meeting of the COP and of its constituted bodies are shown. (**B**) Top: Overall participation of each country (in number of meetings). Middle: Percentage of speeches per country (i.e., ratio between the number of meetings in which a country was a speaker and the number of meetings in which it participated). Bottom: Average stubbornness of each country (mean is over the number of meetings in which a country was a participant). See also figs. S1, S2, and S3. (**C**) Number of meetings per year (leftmost panel) and yearly average number of participants (second from the right), speakers (second from the left), and stubbornness (rightmost). (**D**) Average stubbornness versus participation (left) and percentage of speeches (right) for each party.

The agreement itself is the result of a complex multilateral process of diplomatic negotiations that lasted more than a decade. Central to this negotiation process is the so-called Conference of the Parties (COP), an annual plenary meeting normally held in December. If the COP is the main venue for the important decisions, to deal with the multiple aspects entering into a comprehensive agreement meant to tackle climate change, the UNFCCC has created a number of technical bodies whose role is to help the COP in dealing with specific topics (carbon emission mitigation, adaptation to the effects of climate change, climate finance, green technology transfer, climate agreement implementation, legal and procedural matters linked to climate agreements, etc.). These bodies, called constituted bodies (see Table 1), also meet regularly and contribute to the decisions that are discussed in the plenary COP meetings. Using the official documentation available on the UNFCCC website (https://unfccc.int) and at other repositories such as the Earth Negotiations Bulletin (https://enb.iisd.org/enb/vol12/), we assembled a database containing detailed information on which countries participated to each meeting, and also which countries expressed their views at each meeting, over the period 2001–2015 (see Fig. 1).

**Table 1.** UNFCCC climate change agreement: COP and constituted bodies considered in this study. The number of members of each body, the starting year, and the number of meetings are also shown. The total number of meetings considered in this study is 295. More details on the role of the constituted bodies and their composition are available in the Supplementary Materials.

**Acronym**	**Body**	***n* of members**	**Starting year**	***n* of meetings**
COP	Conference of the Parties	196	2001*	15
AC	Adaptation Committee	16	2012	8
AFB	Adaptation Fund Board	32	2008	26
CTCN	Climate Technology Centre & Network	16	2013	6
CC-E	Compliance Committee - Enforcement Branch	20	2006	27
CC-F	Compliance Committee - Facilitative Branch	20	2006	17
CGE	Consultative Group of Experts	24	2003	24
CDM EB	Executive Board of the Clean Development Mechanism	20	2002	86
JISC	Joint Implementation Supervisory Committee	20	2006	37
LEG	Least Developed Countries Expert Group	13	2002	28
SCF	Standing Committee on Finance	20	2012	11
TEC	Technology Executive Committee	20	2012	10

*The reported starting year refers to our analysis. The first COP was actually held in 1995.

The aim of this paper is to use this dataset to build a dynamical model of this long and complex negotiation process. In particular, the model we propose interprets the negotiation process as a multiagent dynamical system in which the opinions on climate-related issues of the “agents” (i.e., the parties) are the states of the system. During each meeting the participating parties negotiate and influence each other. Meeting after meeting, the opinions progressively get closer to each other and, on the 15-year time scale, manage to achieve a common value [a “consensus” in multiagent language terminology; (*1*)], represented by the Paris Agreement itself. In other words, from a dynamical systems’ point of view, we think of the Paris Agreement as a case of multiparty consensus being achieved through a series of temporal steps, each consisting of a meeting in which various aspects are treated, and propositions are put forward and discussed.

The perspective we take in the paper to describe this process is to consider a dynamical model with two different time scales, in the style of (*2*–*6*): The short time scale is that of the single meeting, lasting 1 to 2 weeks, while the long time scale is spanning the whole 15 years it took for the parties to achieve the unanimous and comprehensive agreement.

Looking at the short time scale, during each meeting, the participating parties discuss some topics of relevance to the convention. Each body is in charge of certain aspects, as listed in the Supplementary Materials. The resolutions approved at the end of the satellite meetings are reported in the next meetings and in the annual COP, where they are treated by the plenary assembly. To model the process of negotiation during a meeting, we use a Friedkin-Johnsen (FJ) model (*7*), i.e., a dynamical model in which the opinions of the participating agents are updated according to a convex combination of the agents’ opinions, and a stubbornness coefficient is associated to each agent to express the fact that different parties tend to defend their positions and to have different degrees of openness or prejudice versus the other parties (*8*). In our context, a natural way to classify parties as stubborn is to browse the minutes of a meeting and find out which parties presented motions to the audience (we call these the “speakers” of a meeting). On this short time scale, the FJ model converges to a point in the convex hull of the initial conditions. If the states of the model represent the opinions of the agents, then the final opinions are closer to each other than the initial opinions, yet they are still normally different from each other, i.e., the agents do not reach an agreement. However, if these opinions are used as the starting point of the next meeting, and the process is iterated, then it is easy to understand that, under suitable conditions, we can expect the opinions to become closer and closer and eventually to converge to a consensus value if the number of concatenated meetings is long enough. The slow time scale of our framework is meant to represent the concatenation of meetings. In this slow time scale, the resulting model can be represented as a product of stochastic matrices, i.e., nonnegative matrices with unitary row sum, in which each stochastic matrix represents the evolution of the opinions during the corresponding meeting. In the simple case in which the interaction graph of each meeting is fixed and constant over the events (i.e., the meetings), it is already known from the literature that by concatenating FJ models in this way, it is possible to achieve consensus (*6*). To properly model the Paris Agreement process, however, the composition of the participating parties must change at every meeting, as only the annual COP meetings are plenary, while the meetings of the constituted bodies have restricted participation, according to complex rotation schemes. Changing the interaction graphs leads, in the slow time scale, to a product of inhomogeneous stochastic matrices, with entries that vary meeting by meeting. For the resulting inhomogeneous backward Markov chain, the convergence analysis is much more complicated and delicate, especially since these stochastic matrices are endowed with special structure and properties that derive from the underlying FJ dynamics occurring at each meeting.

The topic of convergence (or ergodicity) of inhomogeneous Markov chains has been investigated since the 60s (*9*), and many alternative characterizations exist. For instance, for a given sequence of stochastic matrices, a commonly used sufficient condition valid for matrices that have positive diagonal (and hence are aperiodic) is the so-called joint connectivity condition (*10*, *11*), which says that the union of the graphs associated to the stochastic matrices over any interval with some fixed length is strongly connected. Other classes of sufficient conditions include the repeatedly jointly rooted condition (*12*), the sequentially weakly connected condition (*13*), or the cut-balance property (*14*, *15*).

A consequence of the structure inherited from the FJ model is that our stochastic matrices are not SIA (stochastic, indecomposable, and aperiodic) and do not have (fully) positive diagonal, meaning that most of the aforementioned convergence methods do not apply. The condition we develop in the paper for this scope is inspired by the aforementioned literature, and it is based on the idea of trellis graph (*16*), i.e., of mapping the terms of a stochastic matrix into the edges of a graph connecting two consecutive time slices of our opinion states. It is tailored to the specific structure of the Paris Agreement negotiations, in the sense that it relies on the presence of a yearly plenary meeting (the COP) to guarantee that year-to-year products of stochastic matrices have a positive column. The whole inhomogeneous Markov chain becomes, in this way, breakable into intervals corresponding to products of stochastic matrices having a positive column, which converge to consensus over an infinite time horizon.

When applied to real data, the time horizon is necessarily finite; hence, convergence arguments cannot be applied rigorously. Nevertheless, for the 15-year time span we consider in the paper, it is the case that “practical convergence” occurs despite the substantial noise we added when performing our numerical simulations. The noise is intended to represent exogenous modifications of the opinions of the parties, which is realistic to assume to have occurred in a time horizon of 15 years (and have occurred for several countries).

It is worth remarking that while there exists a considerable literature in trying to identify the position and the influence of various countries and negotiation groups on the Paris Agreement, based, among others factors, on analyzing their proposals (*17*–*20*), on interviews with delegates (*21*), on automatic text analysis (*22*), on discourse network analysis (*23*), and on bargaining coalition models (*24*, *25*), to our knowledge, this is the first time that the negotiation process leading to the Paris Agreement is modeled as a dynamical system. It is also (in our knowledge, at least) the first time that a model such as the FJ is used to characterize the behavior of a “real” large-scale multiagent network outside of the sociological experiments in which it was tested already decades ago (*7*, *26*).

The convergence conditions we consider in the paper are essentially of “qualitative” nature, that is, they are based solely on the topology of the interaction graphs associated to the stochastic matrices, but not on the specific values of the numerical entries of these matrices (which are not available for our data). A qualitative interpretation is necessary also for the opinions, which represent real-valued generic quantities not having a precise correspondence to any specific climate-related matter. Given that our aim in this paper is to understand how the organization of the negotiation process can affect the outcome of the negotiations themselves, these limitations are not oversimplifications in the economy of our model.

As a matter of fact, the analysis we perform in the paper gives us insight of both general nature (i.e., on how complex multiparty mediation processes should be organized in order to have chances of success) and on the specific details of the Paris Agreement case study. For instance, the aforementioned conditions for convergence tell us that consensus is achievable only when one or more opinions reach all parties over repeated time intervals. Plenary meetings are instrumental for that, meaning that the formula adopted by the UNFCCC is well suited in this respect. At the same time, a model of the type we are proposing allows to investigate which parameters influence the convergence of the process and which factors may lead to criticality and failure. For instance, in our model, the convergence time increases when the average stubbornness of the agents increases, i.e., the more the parties tend to defend their standpoints, the longer it takes to reach an agreement. This reflects well the reality of the UNFCCC negotiations, which have always been characterized by a high level of conflictuality and by setbacks to the originally planned agenda, which eventually led to the signing of a major accord only in 2015 in Paris instead of, e.g., in 2009 in Copenhagen. Another feature of the model, useful to shed light on the inner workings of a complex multiparty consensus-seeking negotiation process in general and of the Paris Agreement in particular, is that the model provides a natural way to assess the leadership role of the agents during the negotiation process. Specifically, the left eigenvector of the product of stochastic matrices that form the Markov chain has the interpretation of “social power” (*2*, *4*, *8*) of the agents. The evolution of the social power we obtain in our model is largely coherent with the narrative of the UNFCCC negotiation process when it comes to identifying the major players and also how their influence changed over time (*17*–*19*, *27*). This provides an indirect validation of the predictions of the model.

## METHODS

In this section, we describe first the FJ model used for representing a single meeting and then the concatenation of FJ models. Its structure and its graphical representation in terms of trellis graphs are discussed, and a sufficient condition for consensus is provided. The notion of social power is then introduced for the concatenated FJ model.

### Single meeting dynamics

The overall negotiation process we consider is subdivided into a concatenation of meetings, each of which can be seen as a process of mediation among the parties involved. The dynamical model we choose for representing each meeting is an FJ model (*7*), i.e., a DeGroot-like model in which some agents behave stubbornly, in the sense that they defend their positions while discussing with the other parties. If *m* agents participate in a meeting, the FJ model has the following structure

y(t+1)=(I−Θ)Wy(t)+Θy(0), t=0,1,… (1)

where *y* is the *m*-dimensional opinion vector (one variable for each agent), *W* is a row stochastic matrix, and Θ = diag (θ_1_, …, θ*_m_*), with θ*_i_* ∈ [0,1], is a diagonal matrix representing the stubbornness in that meeting of the *m* agents. Stubbornness here means attachment of an agent to its own opinion, represented by the initial condition *y*(0) at the beginning of the meeting (θ*_i_* = 0 means agent *i* is not at all stubborn and θ*_i_* = 1 means a totally stubborn agent). Denoting 𝒢*_W_* the *m*-node graph associated to *W*, then we assume that 𝒢*_W_* is a complete graph, meaning that every represented party is interacting with every other party during a meeting (i.e., each speaker addresses the whole audience). For a nonplenary meeting, 𝒢*_W_* is a subgraph of the full graph representing the ensemble of parties.

Under the assumption that 𝒢*_W_* is a complete graph and that Θ is not a null matrix, the system of Eq. 1 is stable (*8*) and converges to

$$y(\infty )=\underset{t\to \infty }{\text{lim}}y(t)=\mathrm{Vy}(0)$$ (2)

where *V* = (*I* − (*I* − Θ)*W*)^−1^Θ is a row stochastic matrix, see, e.g., (*2*, *8*, *28*) for more details on the convergence properties of FJ models. If 𝒢*_W_* is complete, assuming that all and only the first *u* < *m* agents are stubborn (i.e., θ*_i_* > 0 for *i* = 1, …, *u*, and θ*_i_* = 0 for *i* = *u* + 1, …, *m*), then *V* has the following structure: *V* = [*R*∣0], where *R* is an *m* × *u* matrix of all nonzero entries, i.e., all *m* agents are “reached” by the opinions of the *u* stubborn agents, and the initial opinions of the *m–u* agents that are not stubborn (those with θ*_i_* = 0) do not influence the steady-state values of the opinions [see Supplementary Materials for details in the construction of *V* (particularly the proof of Proposition 2)].

The final opinion *y*(∞) is a point in the convex hull of the initial conditions, co(*y*(0)). In particular, if no agent is totally stubborn (i.e., if θ*_i_* < 1 for all *i* = 1, …, *u*), then *y*(∞) is strictly in the interior of co(*y*(0)), although it is, in general, not a consensus point, i.e., differences in opinion persist (attenuated) at the end of the meeting.

### Overall dynamics: Concatenated FJ models

To represent the entire chain of meetings that led to the Paris Agreement, we treat each meeting of the COP or of any of its constituted bodies as an event on a second, more coarse-grained, time scale *s* and consider a concatenation of FJ models in which the final condition at event *s* becomes the initial condition of event *s* + 1. See (*2*, *6*) for similar related examples of two time scale opinion dynamics models.

For each *s* = 1,2, …, 𝓁, a different FJ model must be built, since both the participating parties and the stubborn participants change with *s*. Let us denote with 𝒱 the set of all possible agents (i.e., the parties participating to the negotiation), with ℳ(*s*) ⊆ 𝒱 the participants at the *s*-th meeting, and with U(*s*) ⊆ ℳ(*s*) the subset of stubborn participants for the *s*-th meeting of cardinality *n*, *m*(*s*), and *u*(*s*), respectively. Consequently, the *m*(*s*) × *m*(*s*) parameter matrices used in Eq. 1 also become event dependent: *W*(*s*) and Θ(*s*) = diag (θ(*s*)), with

$$\theta (s):\{\begin{array}{cc} \theta_{i}(s)>0 & \text{if}\;i\in U(s)\\ \theta_{i}(s)=0 & \text{if}\;i\in M(s)\setminus U(s) \end{array}$$ (3)

Denote *x*(*s*) the opinion vector of the entire set of *n* agents at the end of meeting *s*. We assume that only agents participating in a meeting modify their opinion at the end of the meeting. This means that if an agent *i* participates to the *s*-th meeting, then its opinion is updated; if instead the agent *i* is absent, then its opinion is left unchanged, and the preexisting value is reported. If *y*(*s*, *t*) ∈ ℝ^*m*(*s*)^ is the state at time *t* of the *m*(*s*) agents participating at the *s*-th meeting, then

$$x_{i}(s)=\{\begin{array}{cc} y_{i}(s,\infty ) & \text{if}\;i\in M(s)\\ x_{i}(s-1) & \text{if}\;i\in V\setminus M(s) \end{array}$$ (4)

Using a permutation matrix Π(*s*) to reorder the indices of the agents, the update of Eq. 2 of the opinions of the *m*(*s*) agents participating to the *s*-th meeting can be rewritten in the following matrix form [*I*_*m*(*s*)_= identity matrix of dimension *m*(*s*)]

$$\begin{array}{c} y(s,0)=[I_{m(s)}|0]\Pi (s)x(s-1)\\ y(s,\infty )=V(s)y(s,0) \end{array}$$

from which the expression in Eq. 4 for the state *x*(*s*) containing the opinions of all agents at the end of the *s*-th meeting can be obtained by including the updated values of the opinions of the *m*(*s*) agents that participated to the meeting and by keeping the same opinions for the *n − m*(*s*) agents that were not in that meeting, i.e.

$$x(s)=\Pi {\left(s\right)}^{⊤}[\frac{y(s,\infty )}{\left[0|I_{n-m(s)}]\Pi (s)x(s-1\right)}]$$

By combining the expressions above, the solution of Eq. 1 for the *s*-th meeting can be rewritten compactly as

x(s)=P(s)x(s−1) (5)

with

$$P(s)=\Pi {\left(s\right)}^{T}[\frac{R(s)|0|0}{0|0|I_{n-m(s)}}]\Pi (s)$$ (6)

where in the block matrix in Eq. 6, the first *u*(*s*) agents are stubborn and the first *m*(*s*) agents are those involved in the meeting and that modify their state [*R*(*s*) is *m*(*s*) × *u*(*s*)], while the remaining *n* − *m*(*s*) agents (“absent”) correspond to the identity matrix lower block. *P*(*s*) is also a stochastic matrix.

The concatenation of *k* meetings becomes, therefore, a left product of stochastic matrices

x(k)=P(1:k)x(0)=P(k)…P(2)P(1)x(0) (7)

with each *P*(*s*) having the structure of Eq. 6.

The following is an example of how our matrix *P*(*s*) looks like when agent 1 is totally stubborn (θ_1_ = 1), agent 2 is stubborn with θ_2_ < 1, agent 3 is participating to the meeting but is not stubborn (θ_3_ = 0), and agent 4 is absent from the meeting


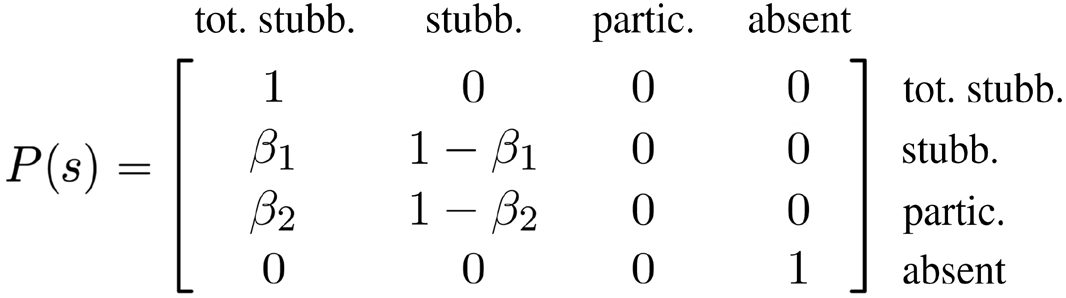
with β*_i_* ∈ (0,1)

The sequence {*P*(*s*)} forms the transition chain of a backward Markov chain {*x*(*k*)}. Backward refers to the use of left products in Eq. 7. Inhomogeneous chains of stochastic matrices such as that of Eq. 7 but without any additional structure have been studied extensively in the literature. It is well known, for instance, that for left products of stochastic matrices such as that of Eq. 7, the condition that *x*(*k*) converges to consensus as *k* → ∞ is equivalent to ergodicity of the chain {*P*(*s*)}, i.e., to require that the infinite product $\underset{k\to \infty }{\text{lim}}P(1:k)$ converges to a rank-1 matrix (*29*)

$$P(1:\infty )=\underset{k\to \infty }{\text{lim}}P(1:k)=1\xi^{T}$$

where **1** is the vector of all 1, and ξ ∈ ℝ*^n^* is a nonnegative vector normalized such that $\sum_{i=1}^{n}\xi_{i}=1$. From this expression, we obtain that the asymptotic value of the opinions in Eq. 5 is

$$x^{*}=\underset{t\to \infty }{\text{lim}}x(k)=P(1:\infty )x(0)=c1$$ (8)

where *c* = ξ*^T^x*(0) ∈ ℝ represents the consensus value achieved by the agents.

The contraction rate of Eq. 7 is given by

$$\rho =\underset{x(0)}{\text{sup}}\;\underset{k\to \infty }{\text{lim}\;\text{sup}}{\left(\frac{\Delta (x(k))}{\Delta (x(0))}\right)}^{\frac{1}{k}}$$ (9)

where $\Delta (x(k))=\underset{i\in V}{\text{max}}\;x_{i}(k)-\underset{i\in V}{\text{min}}\;x_{i}(k)$ is the maximum distance between agents at event *k*. ρ corresponds to the second largest exponent of Eq. 7 [see (*13*)]. By construction, ρ ≥ 0. Convergence occurs when ρ < 1. Small ρ means high convergence rate, while ρ less than 1 but close to 1 means slow convergence (i.e., the convergence rate is $∼\frac{1}{\rho }$).

For the problem we are dealing with in this paper, the transition matrices *P*(*s*) have a special structure and may fail to be SIA. Since *P*(*s*) is not SIA, to get convergence, it is necessary to switch between different *P*(*s*). In fact, from Eq. 6, any time *m*(*s*) < *n*, a block corresponding to the identity matrix appears, hence $\underset{k\to \infty }{\text{lim}}P^{k}(s)$ is not a rank-1 matrix. It is easy to find products of matrices for which convergence to a rank-1 matrix is not achieved, just due to the fact that some agents do not take part to some meetings. Hence, the switching pattern must be designed with some care. Conditions that guarantee the convergence of a Markov chain having the special structure of our concatenated FJ model are not available in the literature but can be derived from it. The one we introduce next is well suited to describe the pattern of the meetings occurring during the UNFCCC climate negotiations, with its alternation of plenary and nonplenary meetings. To understand how consensus can occur, it is useful to use a trellis graph, i.e., a graphical representation of how influences propagate in the concatenation of meetings.

### Trellis graph representation

**Fig. 2.**
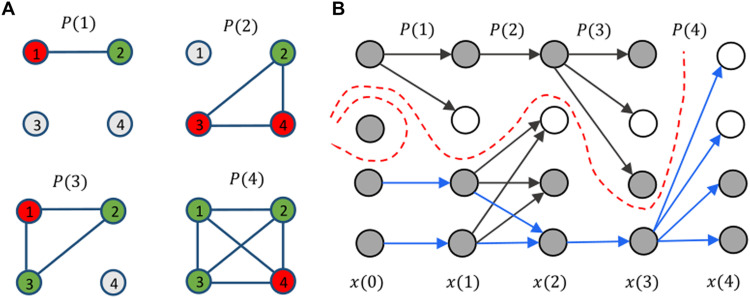
Concatenated stochastic matrices and trellis graphs: An example. (**A**) The four interactions graphs $G_{W}(s)$ corresponding to *P*(1), ..., *P*(4) of Eq. 10. In red, stubborn agents; in green, participating but “silent” agents; in white, absent agents. (**B**) Trellis graph representation of the corresponding backward Markov chain. Each vertical slice represents the state *x*(*s*) of the *n* = 4 agents, and the edges connecting the slice *s* to the slice *s* + 1 are the nonzero entries of *P*(*s*). When a slice *k* becomes reachable from a node (blue paths), *P*(1:*k*) has a positive column. Nodes that turn white are agents that completely lost their initial condition. These correspond to nodes in N(s), i.e., to zero columns in *P*(1:*k*) for some *k*.

The product of stochastic matrices of Eq. 7 can be effectively represented as a trellis graph (*16*), i.e., an *n* × (*k* + 1) directed weighted graph, an example of which is shown in Fig. 2, corresponding to the following four concatenated *P*(*s*) matrices [β*_i_* ∈ (0,1)], representing the four graphs 𝒢*_W_* of Fig. 2A

$$\begin{array}{c} P(1)=[\begin{array}{cccc} 1 & 0 & 0 & 0\\ 1 & 0 & 0 & 0\\ 0 & 0 & 1 & 0\\ 0 & 0 & 0 & 1 \end{array}],P(2)=[\begin{array}{cccc} 1 & 0 & 0 & 0\\ 0 & 0 & \beta_{1} & 1-\beta_{1}\\ 0 & 0 & \beta_{2} & 1-\beta_{2}\\ 0 & 0 & \beta_{3} & 1-\beta_{3} \end{array}]\\ P(3)=[\begin{array}{cccc} 1 & 0 & 0 & 0\\ 1 & 0 & 0 & 0\\ 1 & 0 & 0 & 0\\ 0 & 0 & 0 & 1 \end{array}],P(4)=[\begin{array}{cccc} 0 & 0 & 0 & 1\\ 0 & 0 & 0 & 1\\ 0 & 0 & 0 & 1\\ 0 & 0 & 0 & 1 \end{array}] \end{array}$$ (10)

Each *P*(*s*) represents a step in the trellis graph shown in Fig. 2B. Each node has weighted outdegree equal to 1.

The nodes of the trellis graph are labeled according to two indexes (*i*, *s*), *i* = 1, …, *n*, and *s* = 0,1, …: A vertical group of nodes (indexed by *i*) represents the *n* agents at each event *s*, while the edges between slice *s* − 1 and *s* correspond to the nonzero entries of the corresponding transition matrix *P*(*s*). The special patterns of the trellis graph in our problem are the following:

1) With the exception of the first slice, all nodes must have an incoming edge (*P* has row sums equal to 1).

2) If there is an edge pointing from node *i* to node *j*, then the edge from *i* to *i* also must exist (*R* in Eq. 6 is full).

For each meeting, the possible behaviors of the agents are captured in the trellis graph as follows:

1) Stubborn agent: Node with multiple outgoing edges (one edge for each meeting participant, including self).

2) Participant but not stubborn (“silent”): Node without outgoing edges.

3) Absent: Node with only a self-edge.

Moving from left to right along the trellis graph, it is possible to follow how the opinion of each agent propagates to the other participants in the meetings, according to the rules described above. In turn, opinion propagation can be used to understand when all agents are reached by an opinion and, hence, when a product of stochastic matrices has a positive column.

### Achieving consensus for the Markov chain: Sufficient condition

Denote ℳ(1 : *k*) the cumulative set of parties that have participated to at least a meeting up to event *k*: $ℳ(1:k)={⋃}_{j=1}^{k}ℳ(j)$. Obviously, ℳ(1 : *k*) is nondecreasing: ℳ(1 : *k*) ⊆ ℳ(1 : 𝓁) for 𝓁 > *k*. A first obvious necessary condition for convergence of the Markov chain to a rank-1 matrix is that for certain *k*, ℳ(1 : *k*) = 𝒱. A stronger necessary condition is that *P*(1 : *k*) has a positive column. When this happens, then all products *P*(𝓁)…*P*(*k* + 1)*P*(1 : *k*) also have a positive column, regardless of the choice of *P*(*k* + 1), …, *P*(𝓁). A more formal proof can be given in terms of trellis graphs (see Proposition 1 in the Supplementary Materials). On a trellis graph, having a positive column corresponds to a reachability problem. Denoting ℛ(*i*, *j* : *k*) the set of nodes reached at slice *k* starting from node *i* at slice *j*, i.e., the set of nodes for which there exists a “trellis path” (i.e., a directed path originating at node *i* on the *j*-th slice and traversing the graph from left to right up to the *k*-th slice), then ℛ(*i*,0 : *k*) = 𝒱 for some *k* if and only if *P*(1 : *k*) has a positive column. *P*(1 : *k*) represents the walks of length *k* on the trellis graph initiated at event 0 (see Proposition 1 in the Supplementary Materials for a formal proof). This can be seen in the example of Fig. 2: *P*(1 : *k*) acquires a positive column if and only if an entire slice of the trellis graph is reachable from a node. In this example, *P*(1 : 4) has the third and fourth columns that are positive and correspond to the last slice being reachable from nodes 3 and 4 (see blue paths in Fig. 2B).

The existence of a positive column in *P*(1 : *k*) (i.e., reachability) is, however, not enough to guarantee consensus in the chain of Eq. 7. A simple counterexample is given by prolonging the chain of Fig. 2 with infinitely many *P*(*s*) = *I*. One possible way to obtain a sufficient condition for convergence to consensus is to impose that the reachability property is “renewed” an infinite number of times along the chain. For some integers, *k*_1_ < *k*_2_ < … < *k*_𝓁_ < …, denote *Q*(1) = *P*(1 : *k*_1_), *Q*(2) = *P*(*k*_1_ + 1 : *k*_2_), …, *Q*(𝓁) = *P*(*k*_𝓁 − 1_ + 1 : *k*_𝓁_), … the left products of stochastic matrices in the corresponding intervals [0, *k*_1_], [*k*_1_, *k*_2_],…, [*k*_𝓁 − 1_, *k*_𝓁_], …. If the reachable sets in these intervals, ℛ(*i*_1_,0 : *k*_1_), ℛ(*i*_2_, *k*_1_ : *k*_2_), …, ℛ(*i*_𝓁_, *k*_𝓁 − 1_ : *k*_𝓁_), …, are all equal to 𝒱, then *Q*(1), *Q*(2), …, *Q*(𝓁), … all have a positive column. The Markov chain of Eq. 7 can therefore be compactly written in lumped form as {*Q*(*k*)}. Since each matrix *Q*(*k*) has a positive column, the chain of Eq. 7 converges to consensus, provided that all nonzero entries of *Q*(*k*) remain larger than a given (small) γ > 0 independent of *k*. This can be easily shown for our Markov chain (see Proposition 2 in the Supplementary Materials).

### Social power and loss of influence

In a model such as the concatenated FJ model, it is possible to associate to each agent a notion of social power, describing the relative influence of an agent in the decision process (*2*, *4*, *8*). To compute such influence, notice first that in Eq. 7, when the *k*-th product *P*(1 : *k*) has a zero column, say the *j*-th column, *P*(1 : 𝓁), 𝓁 > *k*, will also have all zeros in the *j*-th column. Denote N(*s*) the set of agents corresponding to zero columns. Then, N(*s*) is nondecreasing N(*k*) ⊆ N(𝓁), 𝓁 > *k*. Also, N can be easily determined on a trellis graph, as it corresponds to some part of the trellis graph being disconnected from the rest and becoming neglected in the trellis propagation. N(*k*) can be formally described as the set of nodes that become unobservable from the *k*-th slice of the trellis graph. An initial opinion *x_i_*(0) is said to be observable at the *k*-th event for some agent *j* if there is a path from (*i*,0) to (*j*, *k*). Denote 𝒪(*j*,0 : *k*) the observable set of initial conditions from node *j* in the first *k* events. Then, *i* ∈ N(*k*) if and only if ${⋃}_{j=1}^{n}O(j,0;k)$ does not contain *i*. For instance, in the example of Fig. 2, *P*(1) already has a zero column (corresponding to node 2), meaning that *x*_2_(0) is completely forgotten by the community already at *s* = 1. Similarly, when computing *P*(1 : 3), the first column also vanishes, meaning that *x*_1_(0) does not also propagate further, i.e., N(4) = {1,2}. The cut sets that partition N(*s*) from the rest of the trellis graph are shown in red dashed line in Fig. 2B. In this case, ${⋃}_{j=1}^{4}O(j,0;4)=\{3,4\}$.

Zero columns in N(*s*) also remain zero when computing *P*(1 : ∞). When consensus is achieved, this implies that the left eigenvector of *P*(1 : ∞) relative to the eigenvalue 1, ξ, has a zero in the *j*-th component. If the Markov chain converges to consensus, then Eq. 8 holds and $c=\xi^{T}x(0)=\sum_{i=1}^{n}\xi_{i}x_{i}(0)$ is a weighted average of the initial conditions *x*(0) with weights given by ξ. In complex network terminology, ξ is the eigenvector centrality associated to *P*(1 : ∞). It is also possible to consider the left eigenvector ψ(*s*) associated to the eigenvalue 1 in the *V*-block of *P*(*s*), which analogously gives the eigenvector centrality for the single *s*-th meeting. For opinion dynamics, ψ(*s*) has the interpretation of instantaneous social power and ξ of (accumulated) social power of the agents: High values in the components of ξ correspond to influential agents in the overall negotiation process, while a zero weight in the *i*-th component means that the *i*-th agent does not have any influence on the final decision (i.e., on the final consensus value *c*), in the sense that the entire community forgets, as *k* → ∞, the initial condition *x_i_*(0).

In terms of meeting structure, three are the possible situations that can lead to a zero column:

1) An agent participates but remains silent in the first meeting [*P*(1) already has a zero column].

2) An agent is either absent or silent in all meetings [i.e., all *P*(*s*) have either a 1 on the diagonal or a zero column in correspondence of the agent].

3) An agent is participating and also active in early meetings, but all the agents who are influenced by it do not propagate their influence in later meetings, i.e., correspond to zero columns in the later meetings (including the agent itself).

In all cases, once an agent has “lost social power” completely (i.e., its column has become 0) at a certain stage, it is impossible to recover it, as in our context, loss of social power corresponds to forgetting the initial conditions, and lost initial conditions are unrecoverable. This property follows from the double time scale structure of the model. Notice that to not lose social power, it is not necessary to behave stubbornly in all the meetings to which an agent participates. For instance, in the example of Fig. 2, agent 3 does not participate in meeting 3; nevertheless, its opinion is carried on by agent 4 who was exposed to it in previous meetings. See also the more complex example in the “A small size example of social power evolution” section of the Supplementary Materials.

### Case of totally stubborn agents

An extreme case for the model of Eq. 7 is represented by a so-called totally stubborn agent. Assuming that one of the agents is totally stubborn for one event means choosing θ*_i_*(*s*) = 1 for some *s*. Then, in Eq. 7, the *i*-th row of *P*(*s*) is the elementary row vector $e_{i}^{T}=[0\;\ldots \;0\;1\;0\;\ldots 0]$. By construction, it is so for the *i*-th row of *I* − (*I* − Θ(*s*))*W*(*s*) and therefore also for that of *V*(*s*). However, unlike an agent that is absent from a meeting, which, from Eq. 6, has both row and column equal to *e_i_*, a totally stubborn agent is influencing the other agents participating to the meeting, i.e., the corresponding column is different from *e_i_*.

If agent *i* is totally stubborn for some meeting, i.e., θ*_i_*(*s*) = 1 for some *s*, but not for all *s*, then convergence can be achieved under the same conditions discussed above. If instead θ*_i_*(*s*) = 1 for all *s*, then *x_i_*(*s*) = *x_i_*(0), i.e., a totally stubborn agent never changes its mind throughout the entire process. If only the *i*-th agent is totally stubborn, then the model of Eq. 7 converges to **1***x_i_*(0), i.e., all agents eventually converge to the opinion of the stubborn agent. If more than one agent is totally stubborn, then no convergence is possible as soon as these totally stubborn agents have different initial conditions.

## RESULTS

### Data collection for the Paris Agreement

The time frame we consider for describing the process that led to the Paris Agreement corresponds to the years 2001–2015 (see Fig. 1). The parties have met since 1995 to discuss the management of climate change and the problems related to it. COP 7 (Marrakech, 2001) marks somewhat the end of the negotiations, leading to the ratification of the Kyoto Agreement and the beginning of a new phase of discussions on future commitments on emission mitigations, with, for example, the creation of several new mechanisms and procedures to manage and coordinate the negotiation process; hence, it was chosen as starting point. The constituted bodies were created at different stages in this time frame and have since met on a regular basis. See Table 1 and Supplementary Materials for details on the bodies, their composition, and their meetings.

By browsing the UNFCCC official documents and/or the minutes of the meetings available on the Earth Negotiations Bulletin (https://enb.iisd.org/enb/vol12/), for each of the 295 meetings in the time frame 2001–2015, we collected data on the participating parties (see data S1 in the Supplementary Materials), which allow us to determine the sets ℳ(*s*) for all *s* (see Fig. 1 and fig. S1). For 63% of these meetings, information could also be gathered on which parties presented arguments to the audience (hereafter the “speakers”) and on how many times each party spoke at a meeting (see Fig. 1 and fig. S2). For meetings in which this information is missing, speakers were assigned randomly. All speaking parties are considered stubborn for the corresponding meeting, with a stubbornness coefficient which is proportional to the number of speeches a party gave at the meeting. In addition, for each meeting, a randomly chosen fraction of the remaining parties is also labeled as stubborn to express the fact that parties may want to defend their opinions even if they do not speak in a meeting. In this way, we obtain the subsets U(*s*) associated to stubborn parties for each meeting. The numerical values of θ*_i_*(*s*) are chosen proportional to the number of times the *i*-th party spoke during the *s*-th meeting when this information is available, randomly when it is not (see Fig. 1 and fig. S3). For each meeting, unless otherwise stated, the values of θ*_i_*(*s*) are rescaled to never exceed 0.95 (i.e., totally stubborn agents are avoided). As for the numerical values of *W*(*s*) [recall that *W*(*s*) is always a full matrix], several choices are possible. Here, we explore three of them:

1) E1: For each meeting, *W*(*s*) are selected at random from a uniform distribution.

2) E2: All weights are identical: *W*(*s*) = **11***^T^*/*m*(*s*).

3) E3: The weights are proportional to the gross domestic product (GDP) of a country: *W*(*s*) = **11***^T^*Φ(*s*), where Φ(*s*) is the diagonal matrix of elements ϕ*_j_*(*s*)= factor proportional to the GDP of the *j*-th country (properly rescaled so that *W*(*s*) is a stochastic matrix).

Here, we report the results for the first weight selection strategy E1, while the other two are discussed in figs. S8 and S9.

### Special structure of concatenated stochastic matrices for the Paris Agreement and “practical” convergence

The central event of the yearly schedule of the UNFCCC program is the annual COP conference. In our notation, plenary attendance corresponds to ℳ(*s*) = 𝒱. If *u*(*s*) is the number of stubborn agents associated to the *s*-th plenary meeting, then the stochastic matrix of a plenary meeting is

$$P^{\text{COP}}(s)=\Pi {\left(s\right)}^{T}[R(s)|0]\Pi (s)$$ (11)

with *R*(*s*) being a full *n* × *u*(*s*) matrix. The interpretation of Eq. 11 having positive columns is that, in a plenary meeting, the stubborn agents exert an influence over all agents.

**Fig. 3.**
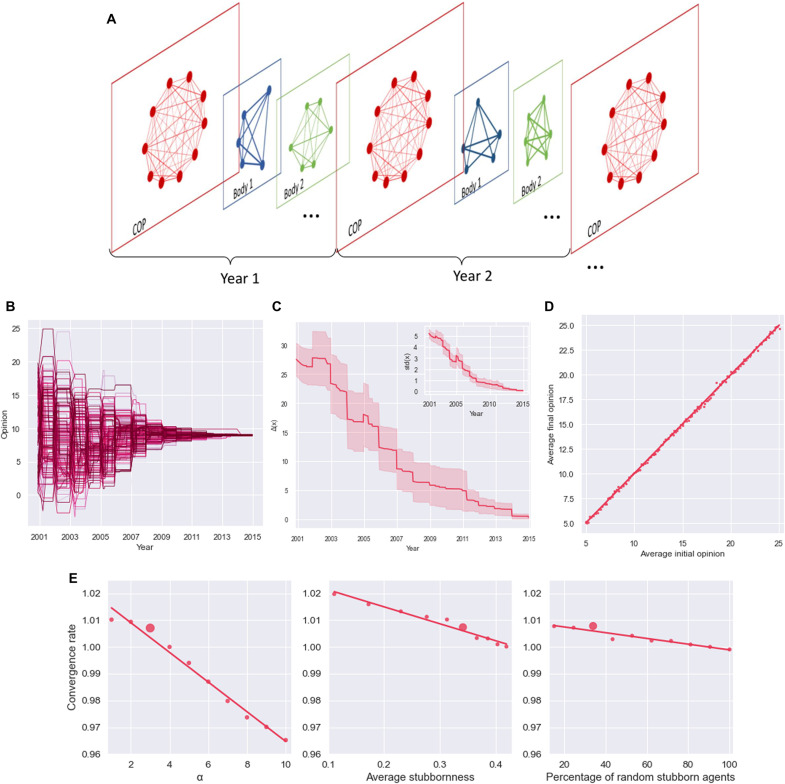
Simulations for the Paris Agreement dynamical model. (**A**) Scheme of the concatenated FJ model used in the paper to describe the achievement of consensus in the Paris Agreement. (**B**) An example of opinion trajectory for the model of Eq. 15 with α = 3. The opinions *x*(*s*) converge to a common value x^*^, i.e., consensus is achieved. (**C**) Behavior of Δ(*x*(*k*)) over time (*k* = years) for the entire set of 10^3^ trajectories, for the same value of α = 3. Inset: same thing but for std (*x*(*k*)). (**D**) Average of the final opinions *x*(15) versus average of *x*(0) as the latter is varied. (**E**) Convergence rate $\frac{1}{\rho }$ as a function of the noise amplitude α (left), of the average stubbornness (computed as the mean over *i* and over *s* of the θ*_i_*(*s*); middle), and of the percentage of extra (i.e., nonspeaking) stubborn agents (right). The solid dot indicates the nominal value used in our simulations.

Let us consider the sequential model depicted in Fig. 3A for representing the concatenation of the meetings of the COP and of the 11 constituted bodies listed in Table 1. Other alternative models are possible, and one is discussed in the Supplementary Materials (see fig. S11). Denote *P^i^*(*s*), *i* = 1, …,11, the stochastic matrix for event *s* associated to the *i*-th constituted body and assume for simplicity of notation that each body meets once a year with a time-order of events that is fixed (the equations can be easily modified to account for different yearly schedules and/or multiple yearly meeting of some of the bodies). Then, with a slight abuse of notation [*P^i^*( · ) indexed by the year rather than by successive events], the concatenation for year *k* is given by the following product (see the sketch in Fig. 3A)

$$Q(k)=P^{\text{COP}}(k)P^{11}(k)P^{10}(k)\ldots P^{1}(k),\;k=2,\ldots ,15$$ (12)

while for “year 1” (i.e., 2002), it is instead

$$Q(1)=P^{\text{COP}}(1)P^{11}(1)P^{10}(1)\ldots P^{1}(1)P^{\text{COP}}(0)$$ (13)

where *P*^COP^(0) corresponds to COP 7 (Marrakech, 2001).

To represent in a more realistic manner the complexity of the process, we decided to allow a certain amount of randomness in the initial condition assumed by each party after each COP. Therefore, instead of the fully deterministic year-to-year update law

x(k)=Q(k)x(k−1) (14)

we consider

x(k)=Q(k)(x(k−1)+ϵ(k−1)) (15)

where for each component ϵ*_i_* of ϵ, it is ϵ*_i_*(*k*) ∼ unif(α · std(*x*(*k*))[ −1,1]) {i.e., ϵ*_i_*(*k*) is a 0-mean value drawn from a uniform probability distribution taking values in [−α · std(*x*(*k*)), α · std(*x*(*k*))], where std(·) is the standard deviation (SD) and α is a positive coefficient}.

The matrices *Q*(1), …, *Q*(*k*) are themselves stochastic matrices but are structured according to Eqs. 12 and 13, to which the convergence conditions developed in Methods can be applied. Assume that no agent is totally stubborn [i.e., that θ*_j_*(*k*) < 1 for all agents], which is reasonable in a context of diplomatic negotiations. The first thing to observe from Eq. 11 is that all *P*^COP^ are characterized by having at least one positive column. Straightforward calculations show that *Q*(*k*) in Eqs. 12 and 13 also have a positive column; hence, the reachability property holds on each yearly interval. Consequently, if, in Eq. 14, we have an infinite product of stochastic matrices (each of them with a positive column), it means that the model of Eq. 14 is converging to a consensus value of the form *c***1** for some constant *c*. Since convergence occurs with an exponential rate, also when Eq. 14 is replaced by Eq. 15, and the time horizon is finite as in our case, we can expect ∥*x_i_*(*k*) − *x_j_*(*k*)∥ to be sufficiently small when ϵ(*k*) is not too large and *k* > 10, i.e., “practical consensus” is achieved. Roughly speaking, this can be quantified in terms of dispersion of the SD along the trajectories: std(*x*(*k*))/std(*x*(0)) is small enough, say <0.1 [which also implies that Δ(*x*(*k*))/Δ(*x*(0)) is small] (see Fig. 3C).

### Numerical simulations for the Paris Agreement data

We used the ℳ(*s*) and 𝒰(*s*) assembled from the data to run 10^3^ simulations of the model of Eq. 15 with {*Q*(*k*)} as in Eqs. 12 and 13 and a nominal value of the external noise amplitude ϵ(*k*) in Eq. 15 corresponding to α = 3. An example of these simulations is shown in Fig. 3B. The addition of the external noise in Eq. 15 renders the process more realistic than in the ideal case discussed in Methods. As can be seen in Fig. 3C, with this choice of noise amplitude, practical consensus is achieved over the 15-year time horizon on the entire set of trajectories. The same also holds true for the weight selections E2 and E3 (see figs. S8B and S9C). As expected by the structure of the model, the final value is an approximate mean of the initial conditions (the added noise has zero mean) (see Fig. 3D).

**Fig. 4.**
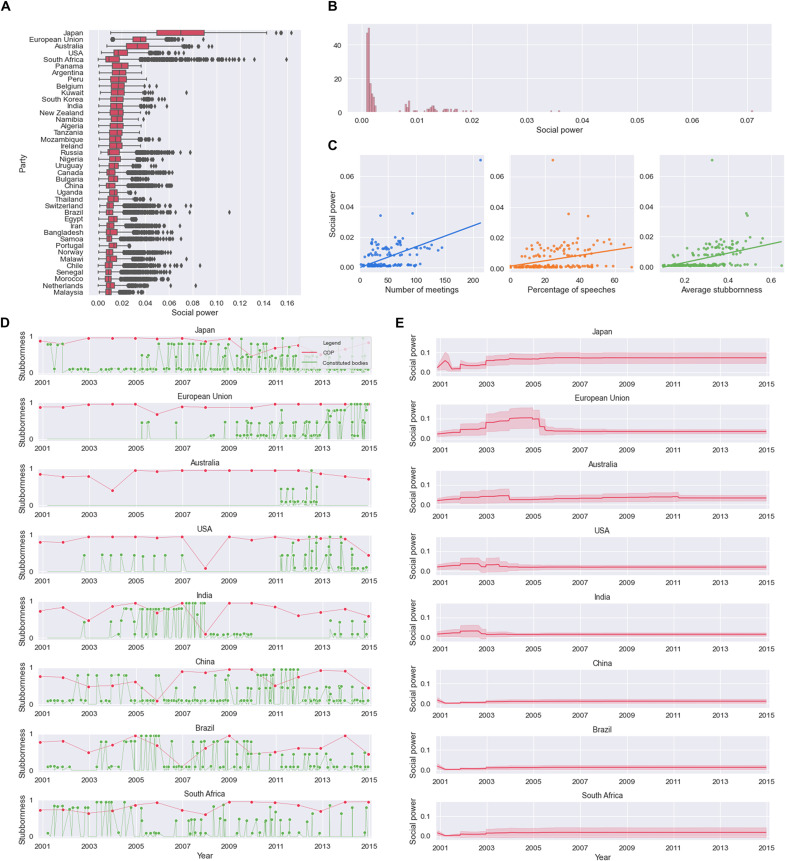
Social power of the parties. (**A**) Box plot of the social power for the 40 most influential parties, over 10^3^ simulations. See fig. S4 for the remaining parties. (**B**) Distribution of social power of all the 196 parties. (**C**) Scatter plots of the social power versus number of meetings per party (left), percentage of speeches per party (middle), and average stubbornness per party (right). (**D**) Time evolution of stubbornness in COPs (red) and constituted bodies’ meetings (green) of eight influential parties. (**E**) Time-dependent evolution of the social power of the same eight parties.

**Fig. 5.**
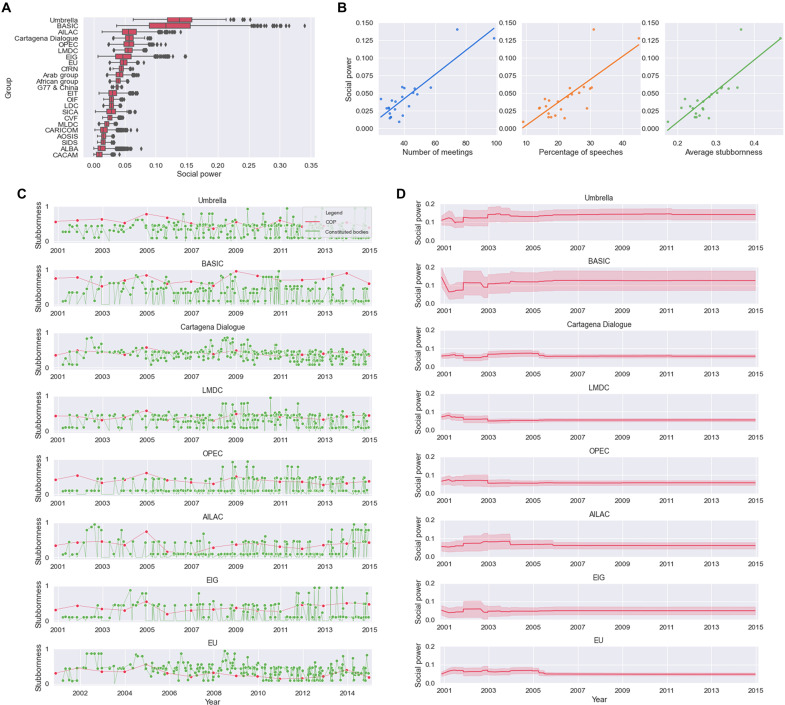
Social power of the negotiation groups. (**A**) Box plot of the social power of the groups over 10^3^ simulations. (**B**) Scatter plots of the social power versus number of meetings (left), percentage of speeches (center) and average stubbornness for each group (right). (**C**) Time evolution of stubbornness in COPs (red) and constituted bodies’ meetings (green) of eight influential negotiation groups. (**D**) Time-dependent evolution of the social power of the same eight negotiation groups.

Recall from Methods that ξ, the left eigenvector of the product of stochastic matrices *Q*(*k*)…*Q*(1), has the meaning of social power accumulated by the agents, i.e., of the relative importance of a party in the final consensus opinion. For the weight selection strategy E1, the social power achieved at the end point in our simulations is shown in Fig. 4. According to our model, a few parties with high social power emerge (see Fig. 4A). See fig. S4 for the other countries (and figs. S8 and S9 for the analogous results on the weight selection strategies E2 and E3). It is useful to investigate which factors determine the social power of a country. In Fig. 4C, the social power of each party is correlated with the number of meetings attended, with the percentage of speeches given at those meetings, and with the average stubbornness of the party. While all three scatter plots show a positive correlation, the presence of many parties with low social power makes the analysis difficult. A more clear picture emerges if, instead of individual countries, we look at the negotiating groups of countries listed in table S2 and compute the social power of a group as the mean of the social powers of the participating countries (see Fig. 5). For instance, from Fig. 5B, we deduce that participation, speeches, and stubbornness are all positive factors in the social power achieved by a group. It is also possible to study how the social power evolves with time. A time-dependent evolution of the social power [obtained by taking the left eigenvector of the product *Q*(*k*)…*Q*(1) as *k* grows] is shown in Fig. 4E at the level of parties and in Fig. 5D at the level of negotiation groups. The time-dependent evolution of the social power of the 40 parties with the highest social power is shown in fig. S6. See Discussion for a thorough analysis.

Several factors influence the convergence rate: the amplitude of the parameter α (i.e., of the uniform noise applied in Eq. 15), the average stubbornness [computed as the mean over *i* and over *s* of the θ*_i_*(*s*)], and the fraction of extra (i.e., nonspeaker) stubborn agents. To evaluate the impact of each of these factors, we performed a sensitivity analysis in which a single parameter is changed at a time, and the corresponding convergence rate is computed as $\frac{1}{\rho }$ (with ρ given by the numerical approximation of Eq. 9). As can be seen in Fig. 3E, the convergence rate shows a negative dependence from all three parameters and has a high sensitivity to the first two (α and the average stubbornness). Notice, particularly, how high values of α compromise convergence (i.e., ρ becomes >1).

**Fig. 6.**
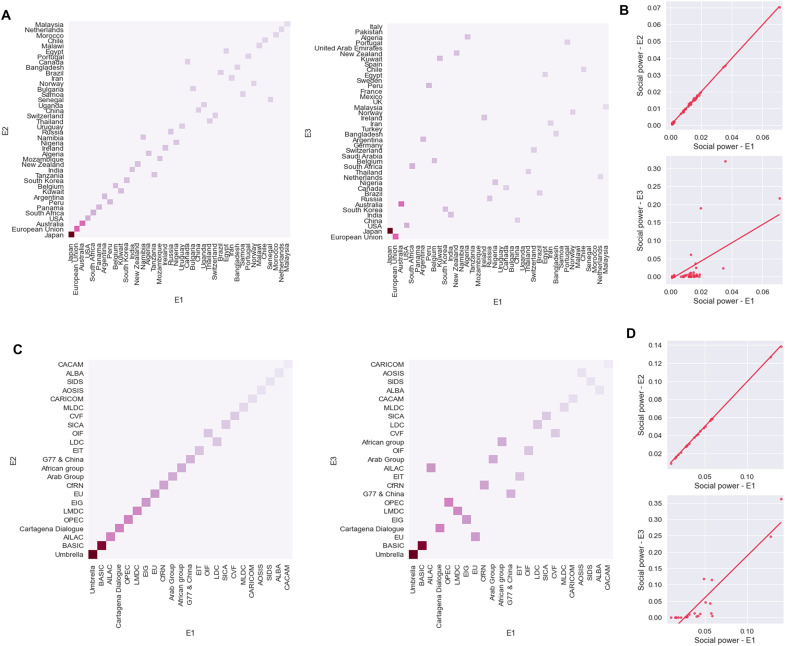
Comparison between the social powers for the weight selection strategies E1, E2, and E3. (**A**) Social power rankings for the 40 most influential parties in E1 versus E2 (left) and E1 versus E3 (right). The color is proportional to the E1 social power. E1 and E2 give very similar social powers, while the ranking for E3 is a bit different (but 29 parties are in common, among the first 40). (**B**) Scatter plots of the social power of the parties in E1 versus E2 (top) and E1 versus E3 (bottom). (**C**) Social power rankings for the groups in E1 versus E2 (left) and E1 versus E3 (right). (**D**) Scatter plots of the social power of the groups in E1 versus E2 (top) and E1 versus E3 (bottom).

The sensitivity of the social power to the choice of weights for *W*(*s*) can be evaluated by comparing the social power rankings given in Fig. 4A, corresponding to the weight assignment strategy E1, with those of figs. S8D and S9A, corresponding to the strategies E2 and E3. Scatter plots of the three different rankings are given in Fig. 6 (A and D). Not only are E1 and E2 very similar, but also E3 has a substantial overlap with E1 and E2. In particular, of the 40 parties having the highest social power, 29 are in common across all three strategies, which is a substantial number. The result is significant at a bootstrapping analysis (see Supplementary Materials and fig. S8C), meaning that the three weight assignment strategies lead to a rather similar social power distribution. This is even more evident if we look at the negotiation groups (see Fig. 6, C and D).

**Fig. 7.**
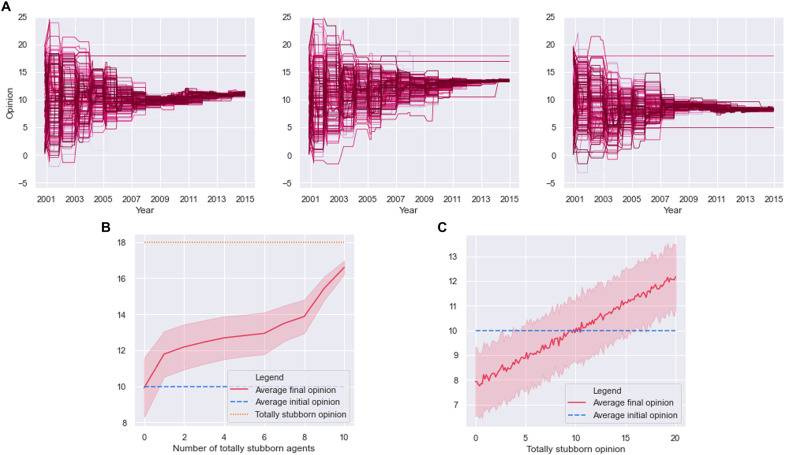
Concatenated FJ model with totally stubborn agents. (**A**) Examples of trajectory for the model of Eq. 15 with α = 3 in the presence of a totally stubborn agent (left), two totally stubborn agents with similar opinions (middle), and two totally stubborn agents with different opinions (right). The straight lines represent the opinions of the totally stubborn agents (which do not change over time). (**B**) Average final opinion of all parties versus number of totally stubborn agents with the same opinion. (**C**) Average final opinion of all parties versus opinion of a single totally stubborn agent.

In our simulations, it is possible to also investigate the case of one or more totally stubborn parties (see Fig. 7). In our model, a single totally stubborn agent for all *s* is able to only slightly steer the opinion of all agents toward its own opinion (see Fig. 7A). If, asymptotically, it is expected that all opinions collapse into the one of the totally stubborn agent, on the finite time horizon we consider, the deviation observed is minor, meaning that the convergence rate in the presence of a single totally stubborn agent is very slow. When two or more parties are treated as totally stubborn, the convergence rate may or may not increase depending on whether the corresponding opinions sit or less on the same side of the average of the initial conditions (see Fig. 7).

## DISCUSSION

Given the enormous political and economical issues at stake, the climate change debate has been characterized by a high level of conflictuality (e.g., North-South divide, disengagement from the accords by some major emitters, obstructions from some major fossil fuel producers, reluctance of developing countries to commit to emission cuts, etc.) rather than by a smooth cooperation among the parties. Using FJ models allows to capture this conflictuality in the form of stubbornness, i.e., of reluctance of the countries to abandon their positions and to mediate an accord. In fact, the main feature of an FJ model is that it allows parties to influence each other [through the connectivity *W*(*s*)] and, at the same time, to defend their positions [through the stubbornness coefficients θ*_i_*(*s*)]. Its main property is that it leads to a contraction in opinion space, which is not as drastic as in a DeGroot model (where consensus is always achieved) but that nevertheless corresponds to an end-point opinion that lies in the convex hull of the initial conditions. In this perspective, it represents well the conflictuality of “constructive” discussion processes, such as diplomatic negotiations, where “some progress” is always made, no matter how small. The validity of the contraction property that characterizes FJ models has been verified experimentally (*26*, *28*), although mostly in small/medium size controlled groups. See also (*30*) for an empirical study over a sequence of issues. An obvious complication to verifying it in more large-scale settings is that it is difficult to assign stubbornness coefficients in a reasonable way. For what concerns our case study, the classical approach from cognitive sociology, namely estimate stubbornness from questionnaires in controlled experiments, or from surveys (*31*, *32*) is not applicable. Also, other elements that might correlate with stubbornness, such as length of a speech or its tone, are ruled out, as speech length is constrained, and “aggressive” verbal attitude (*33*) is not admissible in diplomatic negotiations (and not available from the data). This notwithstanding, our choice of considering speakers as stubborn parties seems actually fairly reasonable, especially in light of the fact that in a COP being a speaker is mostly an opportunity for countries to “posture and stake out their positions” (*27*) on the bargaining table (i.e., a typical manifestation of stubbornness). Taking stubbornness coefficients which are proportional to the number of speeches in a meeting is also coherent with this perspective.

It is worth pointing out that the notion of “consensus” we investigate in this paper is intended as an abstract idea of a long-term multilateral, inclusive, and comprehensive agreement on climate matters and not related to the procedure of “consensus decision” that characterizes the functioning of a COP each year (*27*). More precisely, each COP meeting formally ends with the approval of an accord among the parties, and for that approval, unanimity (or at least lack of formal objections) is normally sought. For instance, famous is the example of COP 15 (Copenhagen, 2009) where negotiations failed, and the proposed text was not adopted, as well as that of COP 16 (Cancun, 2010) where the president “declared” that consensus was reached, although a few members objected. Said otherwise, our approach is not meant to capture the timeline of the forging of the text of the Paris Agreement itself but rather the decade-long process of enabling the near totality of countries on earth to reach a comprehensive agreement.

Recently, different variants of the DeGroot or FJ model have been used to describe opinion dynamics over sequences of issues (*2*–*6*). In most of this literature, however, the issues up for discussion are disjoint, meaning that the initial condition is reset after each discussion, and the aim of the model is to capture how the relative importance of the agents, the so-called self-appraisal, varies over the sequence of discussions (*2*, *4*, *5*). In (*6*), the authors take instead an approach similar to ours: They concatenate the issues being discussed by letting the final state of one discussion to be the initial state of the next one and use the FJ model for every single discussion. The authors show that this form of concatenation can lead to consensus despite each step being characterized by “persistent disagreement.” The paper (*6*) gives also a possible sociological interpretation of the concatenated FJ model in terms of path-dependent theory (*34*, *35*), which says that the decisions one faces are in many circumstances constrained by the decisions one has made in the past. Another interpretation of the concatenated FJ model is that a complex decision cannot be settled in a single meeting by the participating actors but needs to be broken down into many intermediate negotiation steps. These discussions, being part of the same decision process, are typically concatenated, in the sense that the opinions/decisions at the end of a discussion are taken into account in the next meetings, in the spirit of path-dependent theory. These features of a concatenated FJ model resonate well with the reality of climate negotiations, where the issues at stake are very high, the time horizon is sufficiently long, and the multilaterality of the actors involved (the scale being the entire world) makes the negotiation process utterly complex.

The main methodological contribution of this paper is to generalize the concatenated FJ model of (*6*) to time-varying interaction graphs. In particular, what varies in our model is the set of agents participating to each meeting and the set of stubborn agents. Technically, this is similar to extending the DeGroot model from fixed graph to time-varying graphs: The conditions for convergence become much more subtle and mathematically involved (*12*, *13*, *16*). Concatenation in the coarse-grained time scale becomes a product of different (non-SIA) stochastic matrices, and the problem reduces to an inhomogeneous backward Markov chain for which we must find conditions that guarantee convergence to a rank-1 matrix. The sufficient condition we propose in the paper is based on the structure of the so-called trellis graphs associated to the Markov chain (*16*). It is inspired by conditions used for time-varying DeGroot models, but it can deal also with stochastic matrices having an identity diagonal block, as in Eq. 6. This condition is well suited to deal with the case we are investigating in the paper, namely, when we have knowledge of the topology of the interactions at each step and we can reasonably deduce which agents are stubborn, but we do not have any information on the specific coefficients that form the interaction matrix *W*(*s*) nor the stubbornness vector Θ(*s*) at each meeting. An obvious necessary condition to achieve consensus in a time-varying concatenation of meetings such as the one we are considering is that all participating agents should be involved at some stage. This is, however, not a sufficient condition. Sufficiency is achieved if some of the opinions reach all agents [i.e., the reachability condition ℛ(*i*, *j* : *k*) = 𝒱 holds] for sufficiently many time intervals (technically an infinite number of intervals). The organization set up by the UNFCCC, with its annual plenary COP, serves this purpose well, as it guarantees that some influence “percolates” to all parties frequently enough.

The assumption that opinions are concatenated (i.e., that the final state at meeting *s* becomes the initial condition at meeting *s* + 1) is of course a drastic simplification of what happens in real life. The time frame we consider is so long that the policy of a party may change over time because of many factors exogenous to the negotiation process, not least changes of leadership in a country. To cope with this fact, in our simulations, we decided to add a random term to the states after each COP (see Eq. 15). Owning to this term, the concatenation of FJ models is no longer strictly contracting in its convex hull, as can be seen by looking at the simulations of Fig. 3B. Because of this (and because our data cover a finite time horizon), our convergence proof formally no longer applies. Nevertheless, in practice, as long as the amplitude of the noise does not exceed a certain threshold (α < 4, see Fig. 3E), our simulations still consistently show convergence (see Fig. 3C).

Needless to say, the model we present here is a marked simplification of the process being investigated also for several other reasons. First, since the data entered into the model are essentially the participants and the speakers of each meeting, by its own very nature, such a model can represent only the impact of the organization of the negotiation process and not the technical content of the matter being discussed. Second, the opinion of a party on climate issues is represented in lumped form as a scalar real-valued variable (the state of our dynamical system). A more detailed model, in which the opinion of a party is represented as a vector is given in the Supplementary Materials. Even though the variables in this opinion vector are still real-valued numbers, they at least can be used to represent the specific subtopics entering into a climate change global accord (for instance, carbon emission mitigation, adaptation to the effects of climate change, climate finance, green technology transfer, climate agreement implementation, legal and procedural matters linked to climate agreements, etc.). Third, even when focusing on the organization of the negotiation process, we are capturing only (part of) the official, formal part of the process itself. For instance, the constituted bodies listed in Table 1 do not exhaust the auxiliary bodies established to help the functioning of the COP. Several more so-called subsidiary bodies were created over the years, as well as several ad-hoc working groups; see table S1. Both subsidiary bodies and ad hoc working groups meet plenary and normally in conjunction with a COP. For the purpose of our analysis, we decided to disregard these bodies, as the proximity (or sometimes simultaneity) with a COP makes it impossible to disentangle their interactions. Fourth, these extra meetings are only a small part of the myriad of interactions, mostly unofficial, that cannot be captured by our model (or by any other model, for that matter). Most of the negotiations occur behind the scenes and consist of informal contact groups, nonpublic discussions among party delegations, and party group representatives. To give an idea of the scale of the negotiations occurring during a COP, in (*17*), it is reported that COP 18 (Doha, 2012) was attended by more than 4300 party delegates, almost 4000 observers, and more than 600 media participants. The corresponding numbers in COP 21 (Paris, 2015) were 15,000 (+ 8000 “overflow”) delegates, 7000 observers, and 3704 accredited participants from the media.

Nevertheless, despite these limitations and the exclusively “topological” data, it is possible to extrapolate interesting and relevant information on the negotiation process and its dynamics, reflecting trends that are known to have happened for real. For instance, especially for the early years we are considering, the negotiation landscape was mostly characterized by a “North-South” divide, expressed by the Annex I (developed countries) and non-Annex I classification, and the main matter of negotiation was the curbing of carbon emissions of the Annex I countries. The central driving force in this phase was the EU (*21*, *36*), especially because of the disengagement of developed countries such as the United States and Australia from the Kyoto Protocol and the follow-up negotiations on its implementation. In fact, if we look at the time-dependent profiles of the social power of the parties (Fig. 4E), we see that the EU has the highest social power in the years up to 2006. Note that the EU participates to the UNFCCC also as a party, and normally EU states speak as a single voice; hence, the EU as a group, which is an average over the 27 member states, has a lower social power than the EU as a party. The mechanism that leads to this high social power is the same as for agent *a*_1_ in the example of Section 2.1 in the Supplementary Materials: In the early years, the EU participates only to a few meetings (the COPs) and has the highest stubbornness of any other country for those meetings. As the matter under discussion moves from Kyoto and its implementation to post-Kyoto long-term actions, several more actors acquire influence, largely at the expenses of the EU, most notably, the Umbrella group and the BASIC group (see Fig. 5). The rise of the social power of the Umbrella group is to some extent due to the presence of some very active members (in terms of both meeting participation and speeches, e.g., Japan), but also reflects the increased participation of several other countries such as Australia, New Zealand, Russia, and Canada (see fig. S5). The same happens for the BASIC group. The four countries that form this last group (Brazil, South Africa, India, and China) are all large emerging countries, which, in the decade 2001–2010, have seen some of the highest increases in carbon emissions as well as substantial improvements in the quality of life. These countries, together with some of the Umbrella group and oil-rich nations such as OPEC, have expressed over the mentioned decade the strongest resistance to legally binding commitments that could slow their growth (*17*, *37*). Notice that since our information on speeches does not include any sentiment analysis, social power here means both working pro or against an accord. In our model, the balance of leadership tilts around 2006, and it is suggestive to put this in relationship with the significant blow received by the EU agenda in the failed Copenhagen COP 15 (*21*), under the joint opposition of the United States and BASIC countries. Other groups that are mentioned in the literature as having played an important role in the negotiation process, such as for instance AILAC (Independent Alliance of Latin America and the Caribbean) (*19*) and LMDC (Like-Minded Developing Countries) (*17*), are also identified by our model as increasing their social power during the negotiations (see Fig. 5D). For all of these countries and groups, regardless of their political position, assuming a more proactive role in the convention (especially at the level of speeches) can be interpreted as a way to increase their influence on the negotiation table. In our model, this leads to a growing social power. As shown in Figs. 4C and 5B, major factors determining the social power are meeting participation and frequency of the speeches, which in turn (especially the last factor, see Fig. 1D and fig. S7B) drive up the average stubbornness of a country or group. A necessary condition for achieving a high social power in our model is to have a high level of stubbornness, especially in the early years. Owing to their plenary nature, stubbornness in the COP meetings is particularly important, as some influence is transmitted to all parties in just a single step. This is the mechanism that drives up the social power of the EU in the years up to 2005 (see Fig. 4, D and E). In some cases, also maintaining a high stubbornness over many nonplenary meetings can be a fruitful strategy for achieving a high social power, as happens for Japan (see Fig. 4, D and E). In general, however, the time series of stubbornness alone is not enough to deduce the social power of a party. Also, the time-varying topology of *W*(*s*) plays a role, and this can be seen in the number of countries ending with a low social power despite a consistent involvement (cloud of low-lying points in the plots of Fig. 4C). The fact that many countries do not show a linear correlation between social power and participation and/or speaking frequency is a sign that investigating the trellis concatenations in the Markov chains provides valuable information to understand the social power formation and evolution in our model.

In our simulations, the social power, both at the level of parties and groups, tends to stabilize around the years 2008–2010 [see Figs. 4E and 5D (and fig. S6)]. This is due to the product of stochastic matrices *Q*(*k*)…*Q*(1) approaching a rank-1 matrix and is an unavoidable property of our model. If we focus on the “steady-state” social power, i.e., on the values shown in Fig. 4A and fig. S4 for parties and in Fig. 5A for negotiation groups, then the considerations discussed above are still valid and are also, to a good extent, coherent with what is reported in the literature when it comes to describing which parties or groups were the most influential in the overall climate negotiation process (*17*–*21*, *27*, *37*–*39*). We can take this coherence as a form of validation of the outcome of the model, although only an indirect one. Unfortunately, a more classical validation procedure based on independent datasets is not possible in our case, given the type of data that are available and the “uniqueness” of the Paris Agreement negotiation process.

The result remains valid even when we change the weight selection strategy for *W*(*s*). In particular, the ranking of the social powers is, to a large extent, independent of the specific choice of weights in *W*(*s*), at least as long as the matrices *W*(*s*) do not contain any side information. When instead the *W*(*s*) encodes some extra information, some differences can emerge. For instance, including GDP as we do in E3 leads to a higher social power for the rich and the large countries (see fig. S9). Nevertheless, even with this biased choice, the fact that the intersection of the three methods is so consistent (29 parties belong to the top 40 social power rankings in all three weight selection strategies E1 to E3) is a sign that the model is reliable and shows limited sensitivity to parameter uncertainty.

In conclusion, this paper has a twofold objective: On one hand, to propose a dynamical model able to capture and reproduce reasonably well the complex dynamics behind the Paris Agreement negotiation process. On the other hand, a broader goal of this study is to understand how a complex multiparty decision process should be organized to have chances to be successful. The model we develop for this scope, the concatenated FJ model, is tailored to this objective, and its analysis provides criteria that can guide the organization of a complex negotiation process. When applied to the UNFCCC climate negotiations, the approach we have followed appears to be insightful for what concerns the identification of the key parties and their leadership roles in the achievement of the Paris Agreement.

## Supplementary Materials

### This PDF file includes:

Supplementary Materials Supplementary Methods Figs. S1 to S11 Tables S1 and S2 References
