# Supplementary material for: Achieving consensus in multilateral international negotiations: The case study of the 2015 Paris Agreement on climate change
Source: Sci Adv. 2021 Dec 15;7(51):eabg8068. doi: 10.1126/sciadv.abg8068 (PMC8673769; doi:10.1126/sciadv.abg8068)
Supplement: Supplementary file 1 — Supplementary Materials Supplementary Methods Figs. S1 to S11 Tables S1 and S2 References [file sciadv.abg8068_sm.pdf]

Science Advances
AAAS

Supplementary Materials for

Achieving consensus in multilateral international negotiations: The case study of the 2015 Paris Agreement on climate change

Carmela Bernardo, Lingfei Wang, Francesco Vasca, Yiguang Hong, Guodong Shi, Claudio Altafini*

*Corresponding author. Email: claudio.altafini@liu.se

Published 15 December 2021, *Sci. Adv.* **7**, eabg8068 (2021)
DOI: 10.1126/sciadv.abg8068

The PDF file includes:

Supplementary Materials
Supplementary Methods
Figs. S1 to S11
Tables S1 and S2
References

Other Supplementary Material for this manuscript includes the following:

Data files S1 and S2

1 Supplementary materials

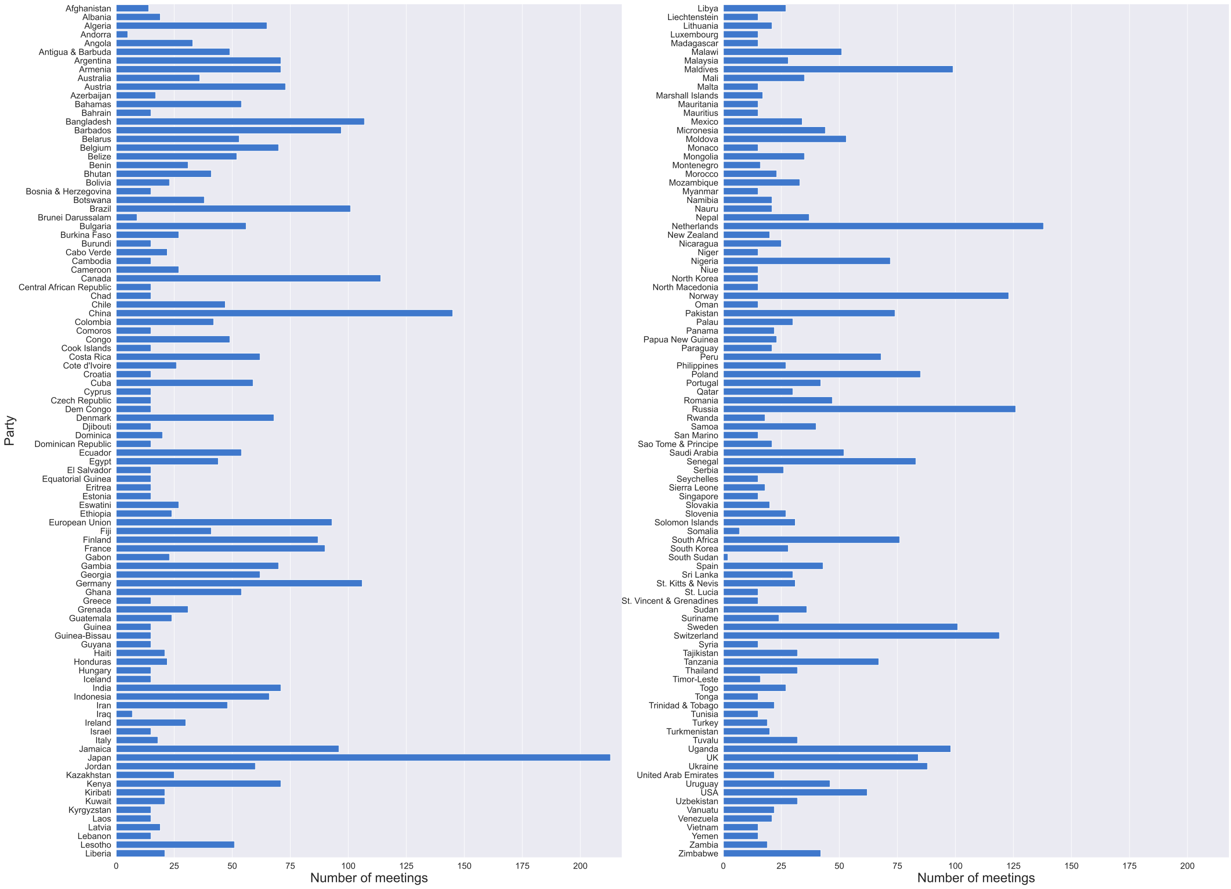

Fig. S1: Number of meetings to which each country participated during the years 2001-2015.

1.1 UNFCCC bodies

The COP is the supreme body of the Convention in which all parties are participants. It first met in 1995, and has met annually since. During the same period as the COP, the Conference of the Parties serving as the meeting of the Parties to the Kyoto Protocol (CMP) meets annually. The first CMP was held in December 2005, in conjunction with COP 11. Over the years, important decisions have been made during COPs. For instance, the Marrakesh Accords made at COP 7 in 2001 consist of the adoption of the Kyoto Protocols rule book with the aim to balance enhanced action to implement the UNFCCC. During COP 15/CMP 5 in 2009, only a voluntary agreement between the parties was reached providing for the reduction of emissions by developed countries and mitigation actions by developing countries. Indeed, the Copenhagen Accord was not formally adopted as an official decision and the key issues were not resolved. In 2010 with the Cancn Agreements adopted in COP 16/CMP 6, major advances on many issues under the UNFCCC,

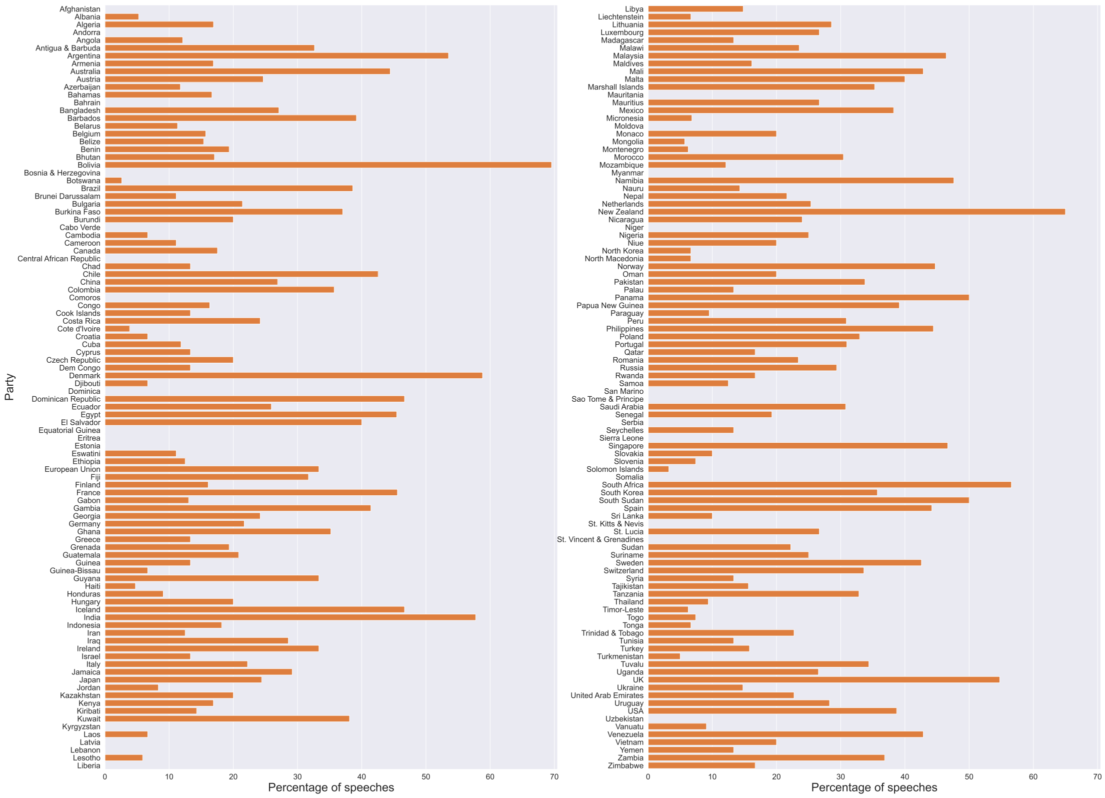

Fig. S2: Percentage of speeches per country (computed as ratio between the number of meetings in which a country was a speaker and the number of meetings in which it was a participant).

including adaptation, technology transfer and finance were reached. During COP 17/CMP 7 in 2011, the Durban mandate was achieved. This decision included establishing a legally binding deal comprising all countries by 2015, which was to take effect in 2020. Moreover, the Paris Agreement was facilitated by the decision taken at COP 19/CMP 9 in 2013 to invite all parties to present their intended nationally determined contributions (INDCs), that is, plans of how they intended to reduce greenhouse gas emissions. By December 10, 2015, 185 countries had submitted their INDCs. Therefore, signing the Paris Agreement meant agreeing to implement the plan previously presented. The Paris Agreement was opened for signature by the parties from April 22, 2016, to April 21, 2017, in New York. 175 parties signed the agreement on the first day it was open for signature. At the time of writing (December 2020), it is not signed by 11 parts of the Paris Agreement (Angola, Eritrea, Iran, Iraq, Kyrgyzstan, Lebanon, Libya, South Sudan, Russia, Turkey, Yemen) yet, while the US has formally withdrawn from the agreement.

In addition to the meetings of the supreme bodies (COP and CMP) mentioned above, other bodies were established to support the parties in the implementation of the Convention and the Kyoto Protocol. In general, the meetings of these bodies are planned before the COP. The active bodies until the Paris Agreement are reported in Supplementary Table S1. These bodies can be classified into three categories: subsidiary bodies, constituted bodies and ad-hoc working groups. The subsidiary bodies meet from 1995 at least 2 times a year in plenary meetings. The subsidiary

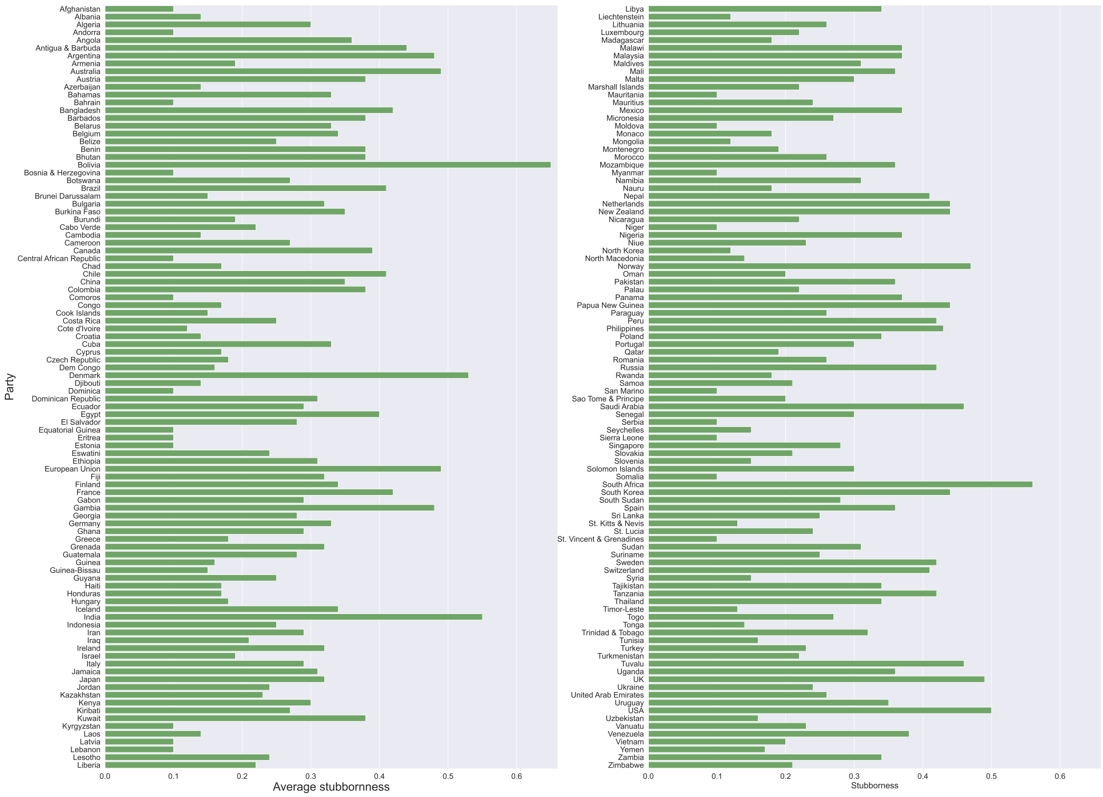

Fig. S3: Average stubbornness per country (computed as mean of the stubbornness coefficients over the number of meetings in which each country was a participant).

bodies are 2:

- the Subsidiary Body for Implementation (SBI) provides relevant guidance and monitors the greenhouse gas emission trends of developed countries, the implementation of the Least Developed Countries (LDCs) work program, the progress on the implementation of the frameworks for capacity-building in developing countries and countries with economies in transition (EITs). Its agenda is shaped around the key building blocks of implementation of all these treaties and instruments: transparency, mitigation, adaptation, finance, technology and capacity-building, and aims at enhancing the ambition of Parties on all aspects of its agenda;

- the Subsidiary Body for Scientific and Technological Advice (SBSTA) provides assessments about scientific knowledge related to climate change and its effects, prepares scientific assessments on the effects of measures decided by the Convention, identifies new technologies, responds to scientific and technological questions from the COP and its bodies. The SBSTA has an important role because it represents a link between the scientific information provided by international organizations, like the IPCC, and the policy needs of the COP.

The constituted bodies meet in sub-group of a fixed number of parties, i.e. their meetings are not plenary. In the time frame considered for our analysis the constituted bodies are 11:

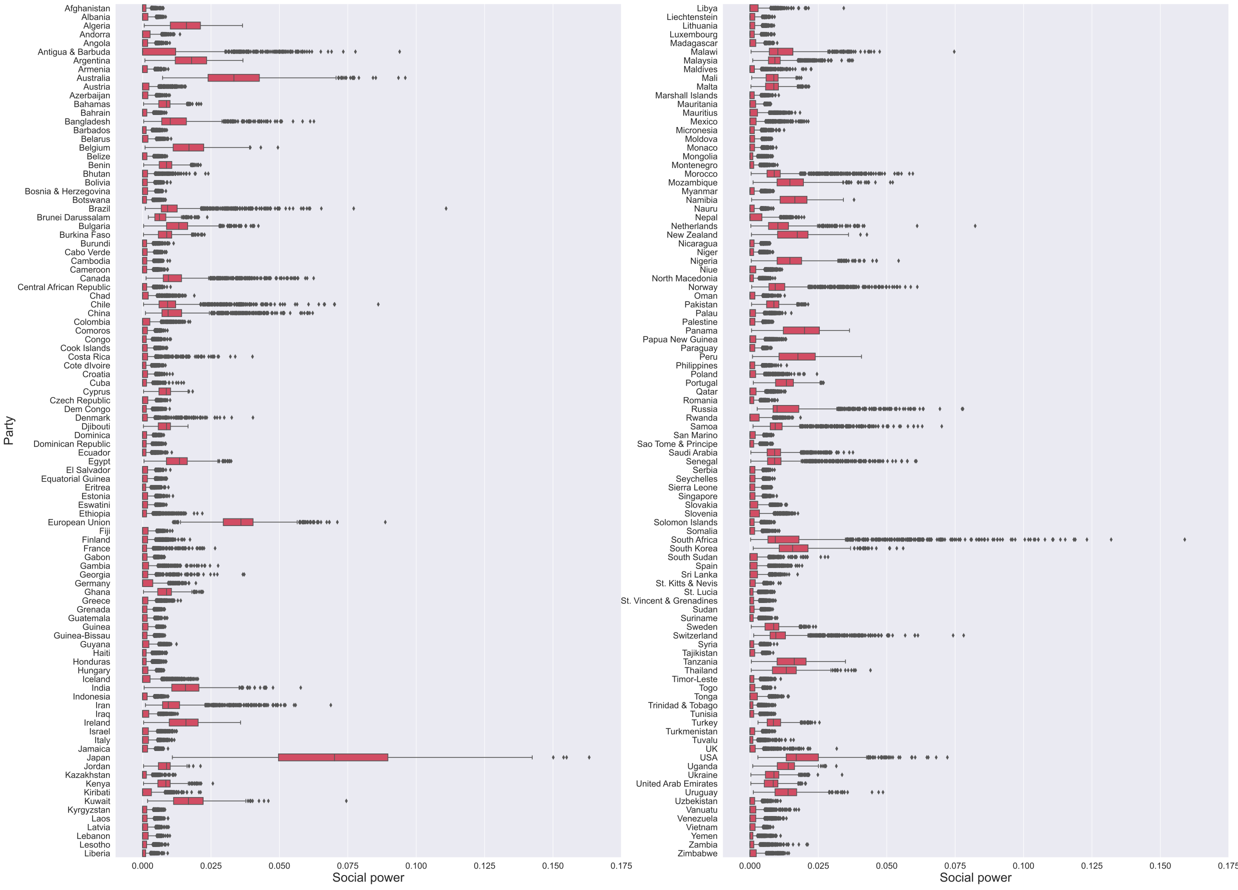

Fig. S4: Box plot of the social power accumulated by each party (over 1000 simulations).

- the Adaptation Committee (AC) met for the first time in 2012 and aims to encourage the development of enhanced actions on adaptation respecting the rules of the Convention, by providing technical support and guidance to the parties, by spreading information, recommendations, experience, knowledge and good practices, by promoting collaboration with national, regional and international organizations. The AC meets at least twice a year and is composed of 16 members, including chair and vice-chair;

- the Adaptation Fund Board (AFB) was established in 2007 with the aim of managing the adaptation fund used to finance projects in developing country parties that are most affected by the effects of climate change. The AFB meetings are planned about 3 times a year. It comprises 16 members and 16 alternates coming from the parties of the Kyoto Protocol of which about 69% from developing countries. Two members serve as chair and vice-chair, with one coming from an Annex I country (industrialized country) and the other from a non-Annex I country (developing country). The position of chair and vice-chair are alternated annually between a member from an Annex I party and a member from a non-Annex I party;

- the first meeting of the Climate Technology Centre (CTCN) was held in 2013. Its mandate

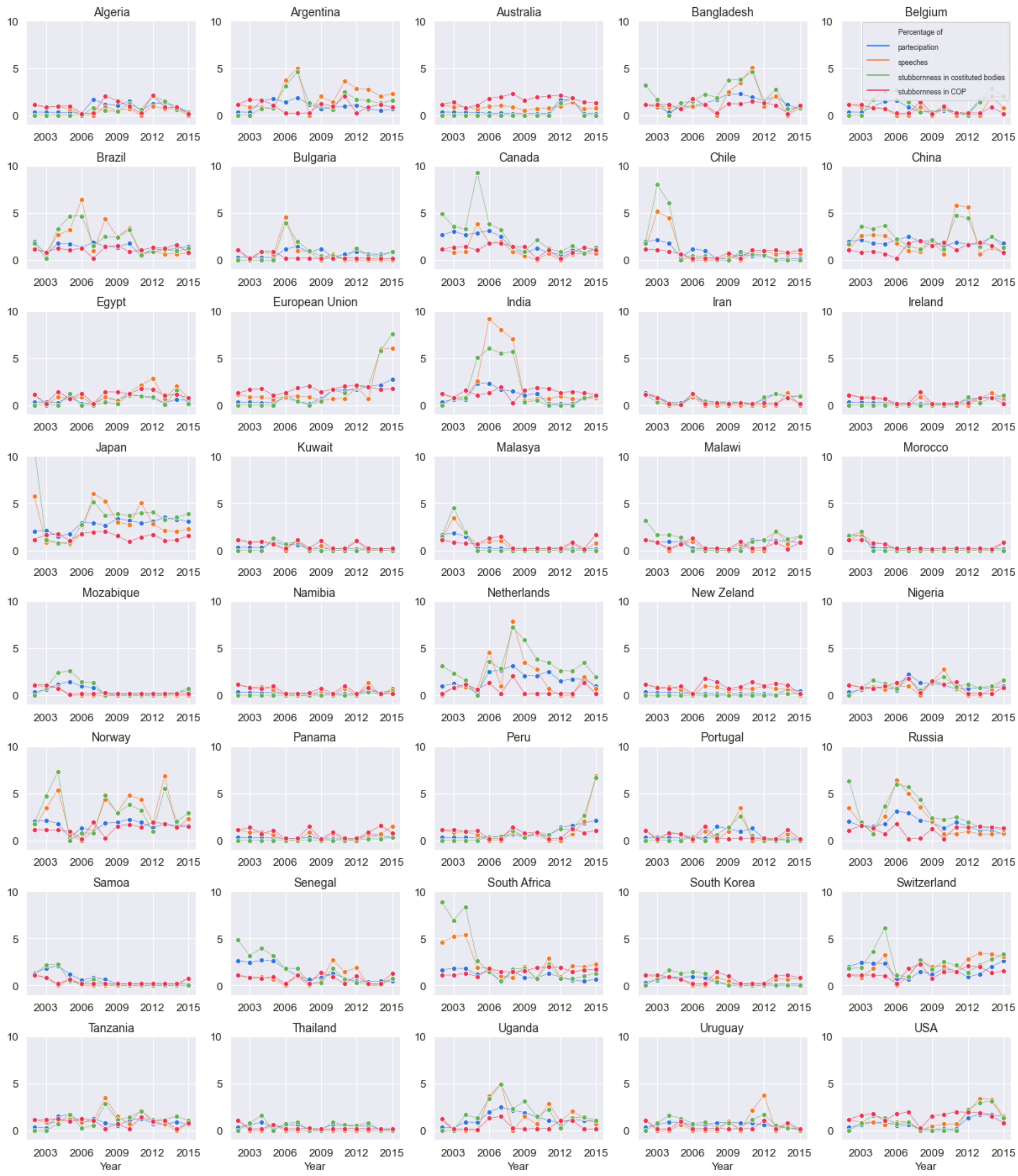

Fig. S5: Time course of yearly participation, speeches, stubbornness in COPs and stubbornness in constituted bodies' meetings for the 40 most influential countries.

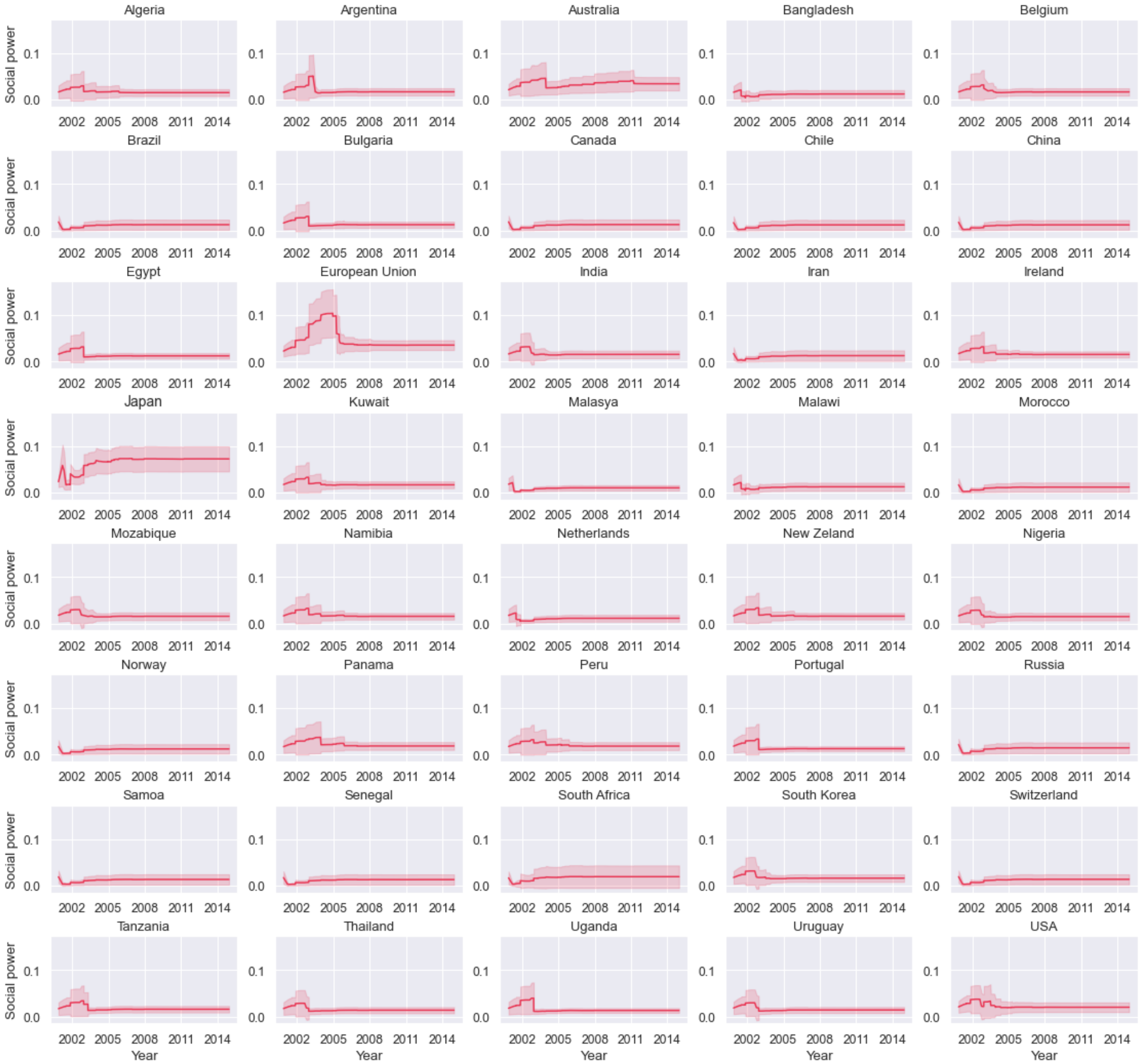

Fig. S6: Social power time course for 40 most influential parties.

is to promote the transfer of environmentally sound technologies for low carbon and climate resilient development in developing countries. In particular, the CTCN, with the support of a stakeholders' network, suggests technology solutions and capacity building regarding the needs of each country. The CTCN meets about 3 times a year. The members are 16, half of them from Annex I countries and half from non-Annex I parties. Moreover, representatives of other subsidiary bodies and UNFCCC observer organization constituencies take part in meetings;

- the CC-E and CC-F constitute the Compliance Committee - Enforcement Branch and Facilitative Branch respectively - of the Kyoto Protocol and they met for the first time in 2006.

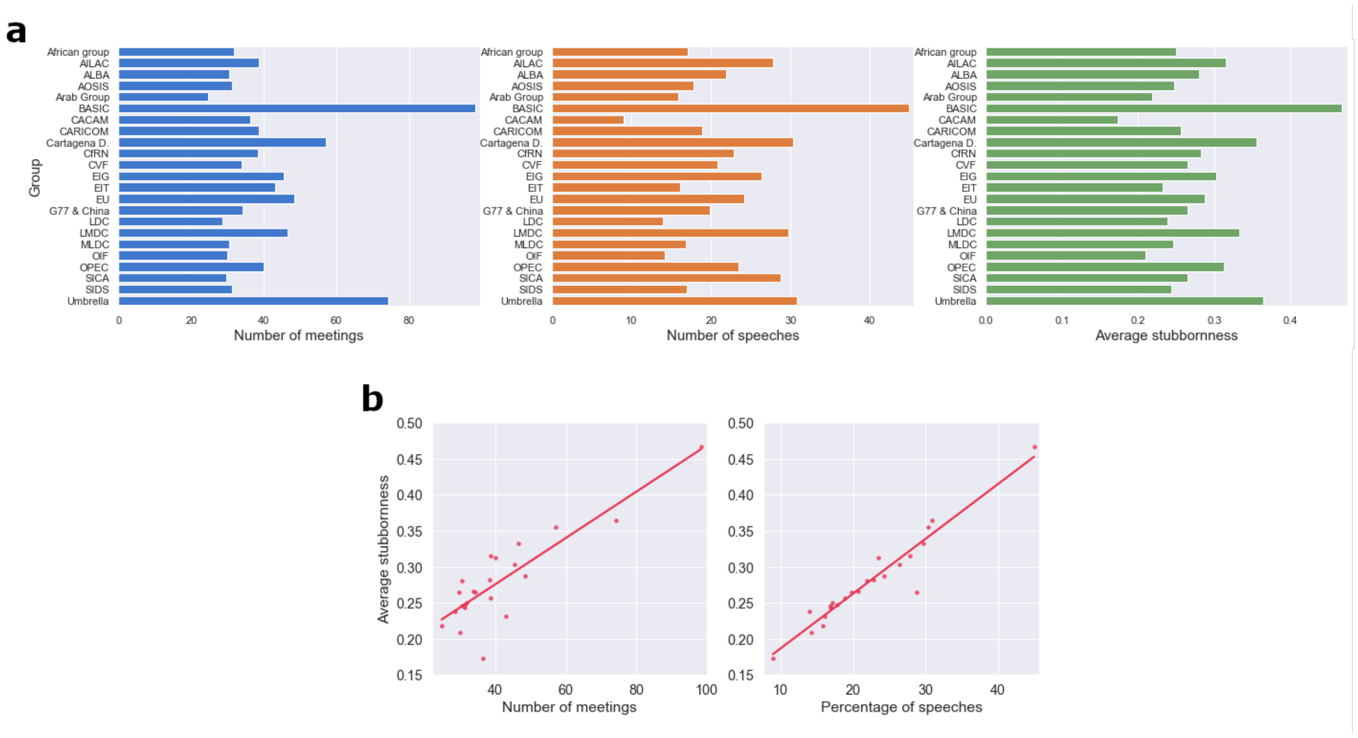

Fig. S7: Data for the negotiation groups. (a): Number of meetings, percentage of speeches and average stubbornness per group. (b): Average stubbornness vs participation and percentage of speeches for each group. All quantities are computed as in Supplementary Figs. S1-S3 and then averaged over the number of parties in each group.

The first one determines consequences for parties that do not follow the provisions of the Kyoto Protocol whilst the second committee provides advice and assistance to parties to respect the Kyoto Protocol. Their meetings are planned about twice a year and in both committees there are 10 members and 10 alternates, including chair and vice-chair;

- the Consultative Group of Experts (CGE) was established in 1999 and is charged with providing technical assistance to developing countries during the preparation of their national communication, i.e., a document presented by each developing country within three years of entering the Convention, and every four years thereafter. Its statute provides two meetings a year plus any additional meeting required to fulfill its responsibilities. The CGE members are 24, including three representatives of international organizations. Two members from non-Annex I countries are chosen as chair and rapporteur. After one year, the chair is substituted by the rapporteur and a new rapporteur is elected;

- the Executive Board of the Clean Development Mechanism (CDM EB) met for the first time in 2001. The CDM EB is the technical committee of the UNFCCC and is responsible to track the activities of the CDM project, i.e., a project that gives credits that can be sold on the market and/or accumulated by private companies or public entities from the developed countries that carry out projects in developing countries aimed at limiting greenhouse gas

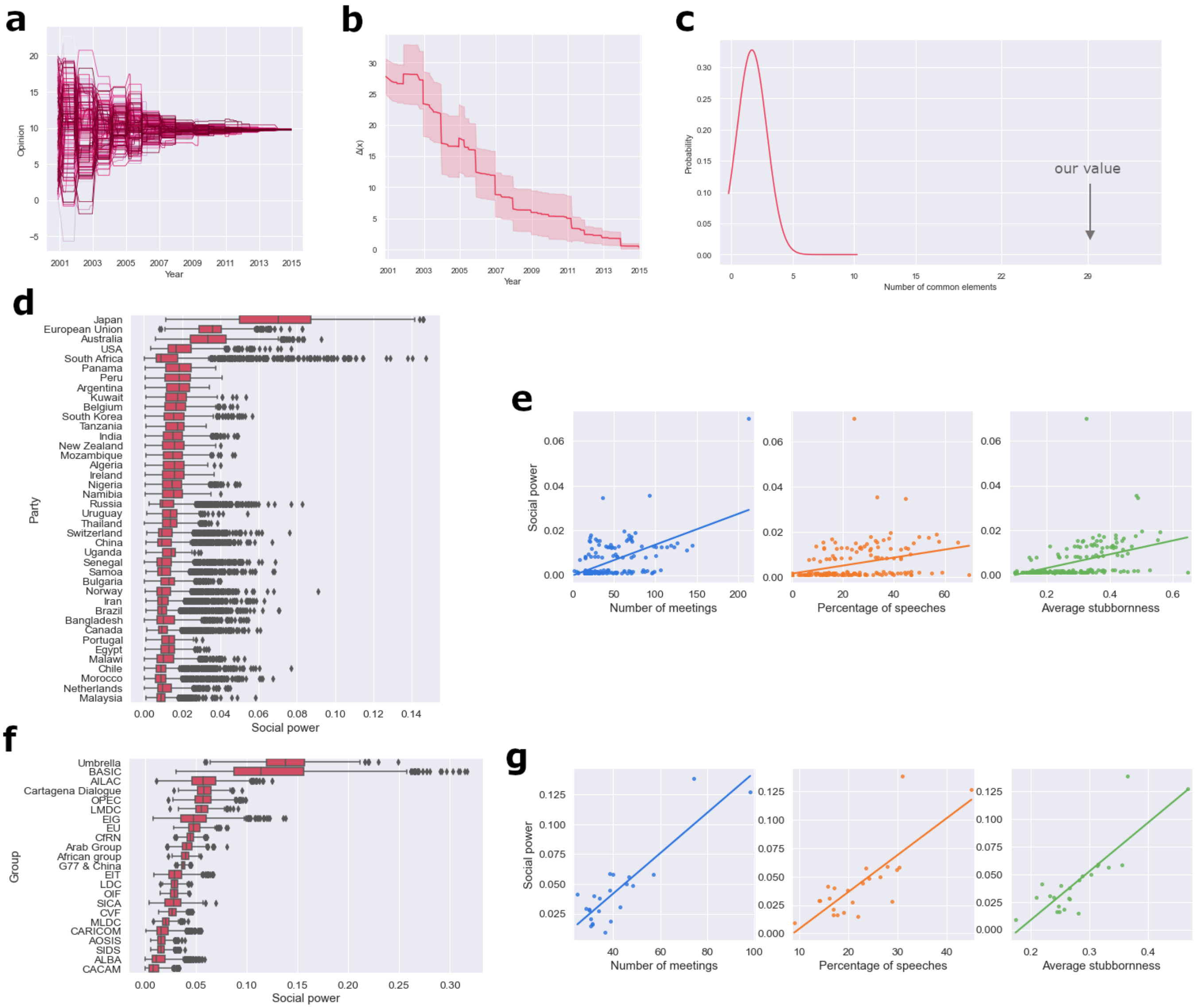

Fig. S8: Simulations for the model with equal-neighbour weights (strategy E2). (a): An example of opinion trajectory for the model with $\alpha = 3$. (b): Behavior of $\Delta(x(k))$ over time (k = years) for the entire set of 10^3 trajectories, for the same value of $\alpha = 3$. (c): Probability distribution for the number of common elements in 3 sets of 40 non-repeating elements chosen at random from 196 possible elements. The whole histogram is much to the left of the value of overlap (29 elements) we get for our E1-E3. (d): Box plot of the social power for the 40 most influential parties, over 10^3 simulations. (e): Scatter plots of the social power of parties vs number of meetings per party (left), percentage of speeches per party (middle) and average stubbornness per party (right). (f): Box plot of the social power of the groups, over 10^3 simulations. (g): Scatter plots of the social power of groups vs number of meetings per party (left), percentage of speeches per party (middle) and average stubbornness per party (right).

emissions. The CDM EB meets about 6 times a year and is composed of 10 members and 10 alternatives. A chair and a vice-chair are elected, with the constraint that one comes from an Annex I country and one from a non-Annex I country;

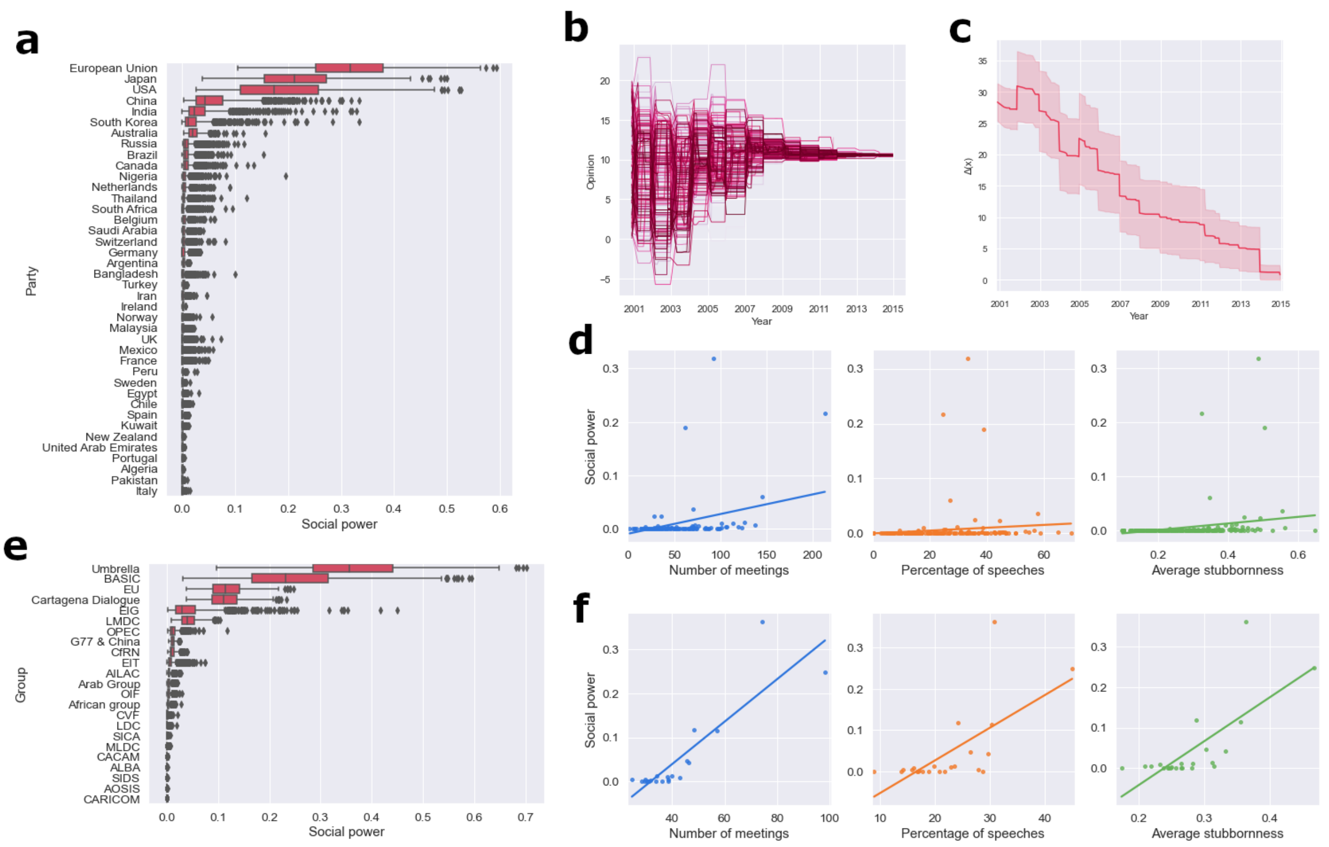

Fig. S9: Simulations for the model with GDP-based weights (strategy E3). (a): Box plot of the social power for the 40 most influential parties, over 10^3 simulations. (b): An example of opinion trajectory for the model with $\alpha = 3$. (c): Behavior of $\Delta(x(k))$ over time (k = years) for the entire set of 10^3 trajectories, for the same value of $\alpha = 3$. (d): Scatter plots of the social power of parties vs number of meetings per party (left), percentage of speeches per party (middle) and average stubbornness per party (right). (e): Box plot of the social power of the groups, over 10^3 simulations. (f): Scatter plots of the social power of groups vs number of meetings per party (left), percentage of speeches per party (middle) and average stubbornness per party (right).

- the Joint Implementation Supervisory Committee (JISC) met for the first time in 2006 and its role is the management of the JI activities, which are projects similar to the CDM project applied for the Annex B parties (sub-group of the Annex I parties that have the emission reduction target). The JISC meets about 4 times a year and comprises 10 members and 10 alternates from parties to the Kyoto Protocol. Each representative can be elected for no more than 2 consecutive terms. The committee nominates annually a chair and a vice-chair, with one being from an Annex I country and the other being from non-Annex I country and with the alternation of the positions of chair and vice-chair between a member from an Annex I party and a member non-Annex I party;

- the Least Developed Countries Expert Group (LEG) was founded in 2001 with the aim of supporting developing countries in implementing national adaptation plans through technical

guidelines, technical papers, training activities, workshops, expert meetings, case studies, capturing and sharing of experiences, best practices. The LEG meets twice a year and comprises 13 members, two of whom are nominated as chair and vice-chair;

- the Standing Committee on Finance (SCF) was established at COP 16 in 2010 to lead the COP to respect the Financial Mechanism of the Convention through some activities, such as the organization of forums to connect bodies and entities dealing with climate change finance, development of guidelines and recommendations relating to the Financial Mechanism of the Convention, and drafting of biennial assessment to track climate finance flows. The SCF meets about 3 times a year and is composed of 10 members from Annex I parties and 10 members from non-Annex I parties;

- the Technology Executive Committee (TEC) was created in 2010 and is mandated to identify policies that allow accelerating the development and transfer of low-emission and climate resilient technologies, by tracking the countries' needs, by promoting actions and guidance, and by creating cooperation among parts and stakeholders. The TEC meetings are held about 3 times a year. The committee is composed of 20 representatives of parties and two members are elected to serve the roles of chair and vice-chair, one from a party included in Annex I and the other from a party included in non-Annex I.

The ad-hoc working groups are bodies born with a specific purpose and to deal with a specific topic. Their work ended when the goal was achieved. Their meetings are plenary. Until the Paris Agreement, 5 working groups have been established:

- the Ad Hoc Working Group on Further Commitments for Annex I Parties under the Kyoto Protocol (AWG-KP) was established during CMP 1 in 2005 to discuss future commitments for developed countries under the Kyoto Protocol. It met for the first time in 2006 and CMP 8 in 2012 decided that the AWG-KP had fulfilled the mandate set out. The AWG- KP met 26 times in formal sessions and one informal session;

- the Ad Hoc Working Group on Long-term Cooperative Action under the Convention (AWG-LCA) was established during COP 13 in Bali in 2007 to define a long-term cooperative action to allow the full implementation of the Convention. It met 22 times from 2008 to 2012;

- the Ad Hoc Working Group on the Durban Platform for Enhanced Action (ADP) was created in December 2011 during COP 17 to develop a protocol applicable to all parties to be adopted at COP 21 in 2015. The ADP met 14 times starting from 2012. Its last meeting was during the first week of the Paris Conference (from 29 November to 5 December 2015) to work on the draft text of the Paris Agreement;

- the Ad Hoc Group on the Berlin Mandate (AGBM) was established by the first Conference of the Parties to implement the Berlin Mandate through actions for the period beyond 2000 by Annex I Parties. It met 9 times from 1995 to 1998;

- the Ad Hoc Group on Article 13 (AG13) was active with 6 meetings from 1995 to 1998 to define how to implement Article 13 of the Convention. Article 13 called for the establishment of a "multilateral consultative process" to help governments overcome difficulties they may experience in achieving their commitments.

It is also reasonable to mention the Intergovernmental Panel on Climate Change (IPCC) in the UNFCCC negotiations. It was created in 1988 under the World Meteorological Organization (WMO) and the United Nations Environment Programme (UNEP). Its aim is to assess climate change science by producing reports in cycles of six to seven years. The IPCC has the role of linking science with policy. Even though IPCC makes its decisions autonomously, the UNFCCC has repeatedly invited the IPCC to prepare reports. Supplementary Table S1 summarizes the key information on each body.

Body	Type	n. of members	Starting year
COP	Supreme	196	1995
CMP	Supreme	196	2005
SBI	Subsidiary	196	1995
SBSTA	Subsidiary	196	1995
AC	Constituted	16	2012
AFB	Constituted	32	2008
CTCN	Constituted	16	2013
CC-E	Constituted	20	2006
CC-F	Constituted	20	2006
CGE	Constituted	24	2003
CDM EB	Constituted	20	2001
JISC	Constituted	20	2006
LEG	Constituted	13	2002
SCF	Constituted	20	2012
TEC	Constituted	20	2012
AWG-KP	Ad Hoc Working Group	196	2006
AWG-LCA	Ad Hoc Working Group	196	2008
ADP	Ad Hoc Working Group	196	2012
AGBM	Ad Hoc Working Group	196	1995
AG13	Ad Hoc Working Group	196	1995
IPCC	External	196	1988

Table S1: Complete list of UNFCCC bodies (plus IPCC). See text for full names and details.

1.2 Negotiation groups

An important role in the UNFCCC climate negotiations has been played by negotiation groups, i.e., groups of countries having common interests and goals that coordinate their efforts and speak with a single voice on one or more topics. In the last decade before the Paris Agreement, the number of groups involved in the negotiation increased, reflecting the complexity of finding a balance between the interests of all parties. In our analysis we have considered the following groups, see also Supplementary Table S2:

- the African group is a regional negotiating group established at COP 1 in 1995 and comprises all 54 African parties;

- the Arabic group is a regional organization with 22 members formed in 1945;
- the European Union (EU) consists of its 28 member states. The EU was established when the Maastricht Treaty came into force in 1993;
- the Group of 77 and China (G77 & China) is a broad coalition of 134 developing parties. It was established in 1964 by 77 countries. China is not a member of Group of 77 but supports it, also financially;
- the Economies in Transition (EIT) group consists of 14 members, which are former Soviet colonies of Russia and Eastern Europe;
- the Least Developed Countries (LDC) group consists of 48 developing nations vulnerable to climate change;
- the Environmental Integrity Group (EIG) was established in 2000 and consists of Mexico, Liechtenstein, Monaco, the Republic of Korea, Switzerland and Georgia;
- the Central American Integration System (SICA) is a regional group of central American countries established in 1990 by Costa Rica, El Salvador, Guatemala, Honduras, Nicaragua and Panama. Subsequently, Belize and the Dominican Republic joined the group;
- the Small Island Developing States (SIDS) is a coalition of 40 developing islands vulnerable to sea-level rise. It was identified as a specific group of developing countries in 1992;
- the Alliance of Small Island States (AOSIS) is an intergovernmental organization of small islands and low-lying coastal states with 39 members and 5 observers born to support the SIDS;
- the Coalition for Rainforest Nations (CfRN) was born in 2005 to promote the collective management of rainforests. It consists of 53 tropical forested nations;
- the Central Asia, Caucasus and Moldova (CACAM) group is made up of 6 countries in Central Asia and Eastern Europe that are not included in Annex I. Some of these parties do not consider themselves as developing countries, therefore they are not members of the G77;
- the Agence intergouvernementale de la francophonie (OIF) is an organization of nations whose population is French-speaking. It was born in 1970 and consists of 54 members, 7 associates and 27 observers;
- the Climate Vulnerable Forum (CVF) is a partnership of 20 countries highly vulnerable to global warming. It first met in 2009;
- the Like-Minded Developing Countries (LMDC) group is made up of 24 developing countries, which represent more than 50% of the world's population. The main objective of this group is to give developed countries greater responsibility for climate change;

- the Mountain Landlocked Developing Countries (MLDC) group was established in 2010 by Armenia, Kyrgyzstan and Tajikistan with the aim of addressing the problems, such as transportation costs and food insecurity, in landlocked mountain developing countries. It includes 16 countries in Africa, 12 in Asia, 2 in Europe and 2 in South America;
- the Organisation of the Petroleum Exporting Countries (OPEC) was formed in 1960 to promote common petroleum policies among its members. Currently, the OPEC has 13 members (Iran, Iraq, Kuwait, Saudi Arabia, Venezuela, Qatar, Indonesia, Libya, the United Arab Emirates, Algeria, Nigeria, Ecuador, Gabon, Angola, Equatorial Guinea and Congo);
- the Umbrella Group is a coalition of 12 non-European Union developed countries (Australia, Belarus, Canada, Iceland, Israel, Japan, New Zealand, Kazakhstan, Norway, Russia, Ukraine and the United States). The main idea of the group is to make the same commitments for the reduction of gas emissions by both developed and developing countries;
- BASIC is the group of 4 large newly emerging economies (Brazil, South Africa, India and China) formed in 2009;
- the Independent Association of Latin America and the Caribbean (AILAC) is a coalition of 8 members (Chile, Colombia, Costa Rica, Guatemala, Honduras, Panama, Paraguay and Peru) established in 2012 with the aim to have a consolidate position in climate change negotiation;
- the Caribbean Community (CARICOM) is an organization established in 1973 to promote economic and cooperation among its members. It consists of 15 full members, 5 associates and 8 observers;
- the Bolivarian Alliance for the Peoples of Our America (ALBA) is a coalition of 9 full members (Antigua and Barbuda, Cuba, Dominica, Grenada, Nicaragua, Saint Kitts and Nevis, Saint Lucia, Saint Vincent and the Grenadines and Venezuela) and 3 observer members (Haiti, Iran and Syria) with socialist ideas;
- Cartagena Dialogue is an informal discussion group born in Cartagena, Colombia, in 2010. It consists of about 40 countries that commit to becoming or remaining low carbon countries.

Supplementary Table S2 summarizes the information about each negotiation group. See also Data S2. Note that some of the groups above were established before the start of the climate change negotiation and might include some nations that are not parties to the Convention.

1.3 Data collection

Our dataset only includes information about the meetings of COP and constituted bodies. In order to compile our database, for each meeting we have collected:

1. the parties participating to the meeting (i.e., the set $\mathcal{M}(s)$ in the notation of the paper);
2. the "speaking parties" (which form a substantial part of the set of stubborn parties $\mathcal{U}(s)$).

Acronym	Group	n. of members
-	African group	54
-	Arabic group	22
EU	European Union	28
G77 & China	Group of 77 and China	134
EIT	Economies in Transition	14
LDC	Least Developed Countries	48
EIG	Environmental Integrity Group	6
SICA	Central American Integration System	8
SIDS	Small Island Developing States	40
AOSIS	Alliance of Small Island States	44
CfRN	Coalition for Rainforest Nations	53
CACAM	Central Asia, Caucasus and Moldova	6
OIF	Agence intergouvernementale de la francophonie	88
CVF	Climate Vulnerable Forum	20
LMDC	Like-Minded Developing Countries	24
MLDC	Mountain Landlocked Developing Countries	32
OPEC	Organisation of the Petroleum Exporting Countries	13
-	Umbrella Group	12
-	BASIC	4
AILAC	Independent Association of Latin America and the Caribbean	8
CARICOM	Caribbean Community	28
ALBA	Bolivarian Alliance for the Peoples of Our America	12
-	Cartagena Dialogue	40

Table S2: UNFCCC negotiation groups.

Concerning the calculation of $\mathcal{M}(s)$, the historical membership of all the constituted bodies is available from the UNFCCC website for the entire period 2001-2015. The minutes of the meetings also contain most of the times the names of the speakers, i.e., representatives of the countries who presented arguments during the meeting, which can be used to infer $\mathcal{U}(s)$. More specifically, we have collected data for each body as follows:

- from the reports of the COP meetings only information about speakers can be extracted. Since the COP is plenary, we have supposed that all parties took part in the meetings;
- the reports of the AC meetings do not contain the list of participants and for 6 meetings they are supported by slides containing the name of speakers. Therefore we have collected the speakers of these meetings and supposed that all AC members were present;
- the reports of the AFB meetings contain information regarding both participants and speakers. Only the first meeting report is not available, and for it we have assumed that all members attended it;
- the CTCN data are the most uneven: for two meetings slides are available from which we could extract the speakers but not the participants, for three meetings we could collect both

types of information based on reports, and no resources relative to the last meeting are available on the UNFCCC website. For the four meetings without the list of participants we have assumed that all members attended them;

- the reports of the meetings of CC-E, CC-F and LEG only contain the list of participants;
- for half of the CGE meetings only the list of participants can be extracted from the reports, while for the other half no reports or slides are available, therefore we have assumed all members participated;
- from the reports of the meetings of CDM EB, JISC and SCF, information about both the participants and the speakers is available;
- concerning the TEC meetings, we could only obtain from the UNFCCC website the list of participants to the last four meetings, therefore for the remaining six meetings we have assumed that all members attended them.

Obviously, we are not interested in the identity of the representatives, but only in the country they represent. Hence participants and speakers are always associated to their country in computing $\mathcal{M}(s)$ and $\mathcal{U}(s)$. With the assumption that all members have attended the meetings for which the list of participants is not available, we have complete data (participants and speakers) for 185 meetings and the list of participants for the remaining 110. For the latter meetings, we have chosen randomly the parties to be treated as stubborn. As mentioned in the paper, also for the 185 meetings in which we have complete information we have considered a fraction of non-speakers as stubborn, i.e., $\mathcal{U}(s)$ is formed by combining speakers and some randomly chosen non-speaker participants.

1.4 Data description

The two Excel files `Data_S1.xlsx` and `Data_S2.xlsx` contain the datasets for the UNFCCC meeting leading to the 2015 Paris agreement (participants and speeches) and the UNFCCC negotiation group, respectively.

1.4.1 Data S1

Each worksheet corresponds to one of the UNFCCC bodies considered in the paper:

- COP (Conference of the Parties),
- AC (Adaptation Committee),
- AFB (Adaptation Fund Board),
- CTCN (Climate Technology Centre & Network),
- CC-E (Compliance Committee - Enforcement Branch),
- CC-F (Compliance Committee - Facilitative Branch),

- CGE (Consultative Group of Experts),
- CDM EB (Executive Board of the Clean Development Mechanism),
- JISC (Joint Implementation Supervisory Committee),
- LEG (Least Developed Countries Expert Group),
- SCF (Standing Committee on Finance),
- TEC (Technology Executive Committee).

The first column contains the list of parties, while the others represent the participation of the corresponding party at the meeting which was held at the date reported in the first row. Each entry can be: 0 (no participation), 1 (participation without speech) and z (> 1, participation with $z - 1$ speeches).

1.4.2 Data S2

The first column contains the list of parties, while the others define the affiliation of the corresponding party to the negotiation group reported in the first row. Therefore, each entry can assume two values: 1 (member) and 0 (no member).

2 Supplementary methods

2.1 A small size example of social power evolution.

The following example is useful to understand how the social power evolves with the stubbornness in a sequence of meetings. Consider 6 agents, denoted by a_i with $i = 1, \dots, 6$, and 15 meetings s_j with $j = 1, \dots, 15$. The stubbornness of the participants is given by the following matrix

$$
\begin{array}{c}
\\ a_1 \\ a_2 \\ a_3 \\ a_4 \\ a_5 \\ a_6
\end{array}
\begin{array}{c}
\begin{array}{ccccccccccccccc}
s_1 & s_2 & s_3 & s_4 & s_5 & s_6 & s_7 & s_8 & s_9 & s_{10} & s_{11} & s_{12} & s_{13} & s_{14} & s_{15}
\end{array} \\
\left[\begin{array}{ccccccccccccccc}
* & 0.9 & * & * & * & * & * & * & * & 0.9 & * & * & * & * & 0.9 \\
0.7 & 0.9 & 0.7 & 0 & 0.1 & 0 & 0 & 0 & 0 & 0.9 & 0 & 0.2 & 0 & 0 & 0.9 \\
0 & 0 & 0.1 & 0.2 & 0 & 0 & 0 & 0 & 0.2 & 0.9 & 0.9 & 0.9 & 0.9 & 0.9 & 0.9 \\
* & * & * & 0.9 & 0.9 & 0.9 & 0.3 & 0.2 & 0 & 0 & 0 & 0 & 0 & 0.1 & 0 \\
0.4 & 0.4 & 0.4 & 0.4 & 0 & 0.4 & 0.8 & 0.8 & 0.8 & 0.3 & 0.4 & 0.2 & 0.1 & 0 & 0 \\
0.9 & * & 0.9 & 0.9 & 0.2 & 0.1 & 0 & 0 & 0 & 0 & 0 & 0 & 0.1 & 0 & *
\end{array}\right]
\end{array}
$$

where the (i, j) entry indicates the stubbornness of agent a_i at meeting s_j and $*$ denotes absence from the meeting. The time course of the social power of each agent, shown in Supplementary Fig. S10(b), is highly dependent on the stubbornness. Compare for instance agents a_1 and a_2. Agent a_1 participates in only three meetings with high stubbornness in all of them, while agent a_2 attends the same meetings in which a_1 is a participant with identical stubbornness but also all other meetings with lower stubbornness. The social power accumulated by agent a_1 is much higher than that of agent a_2, meaning that being a meeting participant with low or zero stubbornness

leads to bigger losses of social power than being absent. The zero social power of agent a_3 in Supplementary Fig. S10(b) derives from the fact that it attends the first meetings with zero stubbornness which implies the third column of $P(1)$ is a zero column and remains zero for all $P(1:k)$ with $k = 2, \ldots, 15$. Moreover, even though the average stubbornness of agent a_5 is greater than that of agent a_2, agent a_2 gains more social power than a_5 due to its high stubbornness in the first meetings, which is not totally lost over time. Finally, observe that although agents a_4 and a_6 have similar profiles regarding stubbornness, apart from a time shift, their social power time courses are very different, showing that the social power is not necessarily correlated with stubbornness.

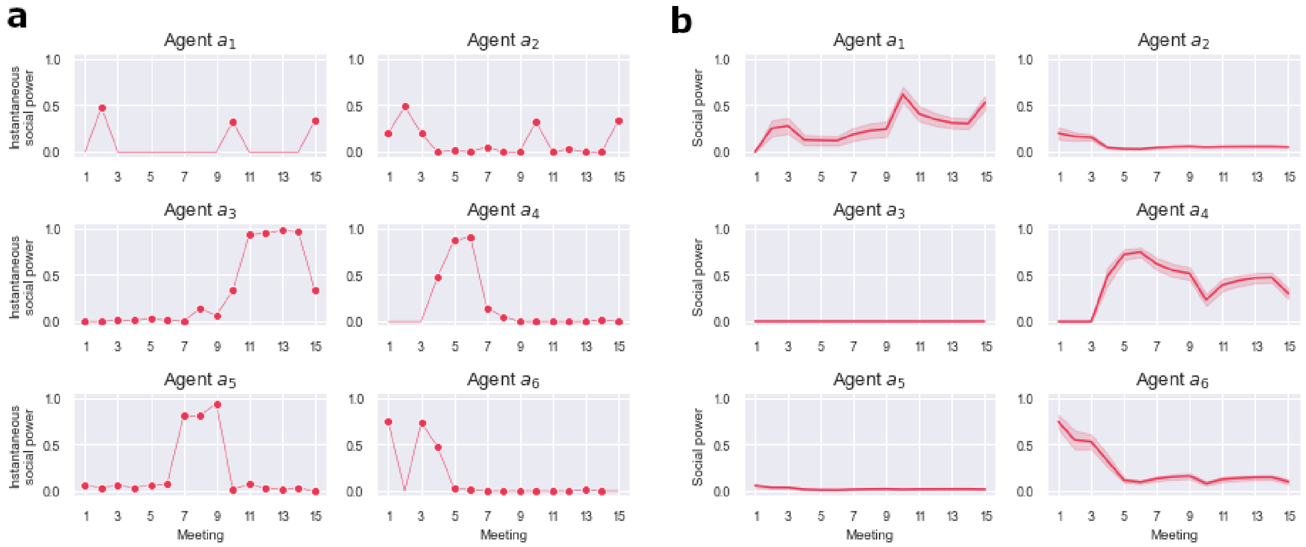

Fig. S10: Social power for the small size example of Section 2.1. (a): Instantaneous social power time course of agents a_i for all $i = 1, \ldots, 6$. (b): (Accumulated) social power time course of agents a_i for all $i = 1, \ldots, 6$.

2.2 Overlap of social power rankings: bootstrapping analysis

To evaluate if the overlap between the social powers we obtain in correspondence of the 3 weight selection strategies E1-E3 for $W(s)$ is statistically significant, we performed a bootstrapping analysis, consisting in randomly choosing 10^6 triples of 40 non-repeating elements in a set of 196 elements, and computing the intersection of each triplet. The resulting histogram, shown in Fig. S8(c), is much to the left of the value of 29 elements we obtained for the overlap of the 3 sets of parties in the top-40 social powers ranking of the E1-E3 strategies (shown in Fig. 6(a) of the paper, and Figs. S8(d), S9(a)), meaning that the E1-E3 overlap is highly significant (P-value is 0).

2.3 Theoretical analysis of the model

In this section, we provide a more formal analysis of the convergence of the concatenated FJ model described in the paper. The analysis is inspired by analogous conditions on time-varying DeGroot

models (*40, 41*), with the extra feature that our stochastic matrices $P(s)$ have the structure shown in Eq. 6 of the paper (in particular a diagonal block equal to the identity). As in the main manuscript, split $\mathbb{N}$ into the bounded intervals $[0, k_1], [k_1+1, k_2] \ldots, [k_{\ell-1}+1, k_\ell], \ldots$ and denote

$$Q(1) = P(1 : k_1), \;\; Q(2) = P(k_1+1 : k_2), \;\ldots,\; Q(\ell) = P(k_{\ell-1}+1 : k_\ell), \ldots \tag{1}$$

the left products of stochastic matrices in those intervals.

Proposition 1 *Consider the Markov chain of Eq. 1.*

1. $\mathcal{R}(i, k_{\ell-1} : k_\ell) = \mathcal{V} \iff$ *the i-th column of* $P(k_{\ell-1}+1 : k_\ell)$ *is positive;*
2. *if* $P(k_{\ell-1}+1 : k_\ell)$ *has a positive column for some* k_ℓ*, then* $P(k_{\ell-1}+1 : r)$ *has a positive column for all* $r > k_\ell$*.*
3. *A necessary condition for consensus is that* $\exists\, i \in \mathcal{V}$*,* $k_{\ell-1}, k_\ell \in \mathbb{N}$ *such that* $\mathcal{R}(i, k_{\ell-1} : k_\ell) = \mathcal{V}$*.*

Proof.

1. By construction, the columns of $P(k_{\ell-1}+1 : k_\ell)$ represent all paths of length $k_\ell - k_{\ell-1}$ between pairs of nodes. Reachability from i on the trellis graph means that $\exists$ a path of length $k_\ell - k_{\ell-1}$ from i to all nodes.
2. Consider the reachable set for i, $\mathcal{R}(i, k_{\ell-1} : k_\ell)$. If $\mathcal{R}(i, k_{\ell-1} : k_\ell) = \mathcal{V}$, then necessarily also $\mathcal{R}(i, k_{\ell-1} : k_\ell + 1) = \mathcal{V}$ since each node has to have at least one incoming edge and all edges on the k_ℓ-th slice are reachable from i. This proves that if $P(k_{\ell-1}+1 : k_\ell)$ has a positive column, so does $P(k_{\ell-1}+1 : r)$ for all $r > k_\ell$.
3. If $\mathcal{R}(i, k_{\ell-1} : k_\ell) = \mathcal{V}$, then $P(k_{\ell-1}+1 : k_\ell)$ has a positive column and, as we have just shown, also any other infinite product

$$\lim_{r\to\infty} P(r) \ldots P(k_\ell+1) P(k_{\ell-1}+1 : k_\ell) \tag{2}$$

has a positive column. If Eq. 2 does not have any positive column, then it cannot be a rank-1 matrix.

■

Notice that condition 3 of Proposition 1 is necessary but not sufficient for the limit (2) to converge to a rank-1 matrix: if $P(k_{\ell-1}+1 : k_\ell)$ has a positive columns and it is concatenated with ∞-many $P_1 = \left[\begin{array}{c|c|c} R & 0 & 0 \\ \hline 0 & 0 & I_{n-m_1} \end{array}\right]$, then $\lim_{r\to\infty} P_1^r P(k_{\ell-1}+1 : k_\ell)$ is not a rank-1 matrix. In order to obtain a sufficient condition, the interval $[k_{\ell-1}+1 : k_\ell]$ in which $P(k_{\ell-1}+1 : k_\ell)$ has a positive column has to be "renewed" ∞-many times, see Theorem 1 below. Before stating it, we need the following technical condition.

Proposition 2 *Consider the Markov chain of Eq. 1. Assume that*

- *there exist 2 sequences* $\{k_\ell\}$ *and* $\{i_\ell\}$, $k_\ell \in \mathbb{N}$, $k_0 = 0$, $k_{\ell-1} < k_\ell$, $i_\ell \in \mathcal{V}$, *and an integer* $\kappa > 0$ *such that* $k_\ell - k_{\ell-1} \leq \kappa$*;*
- *there exists* $\mu_1 > 0$ *such that all positive entries of* $W(s)$ *and, if any, of* $\Theta(s)$ *are lower bounded by* μ_1 *for all* $s \geq 1$*. In addition, there exists* $0 < \mu_2 < 1$ *such that all entries of* $\Theta(s)$ *are upper bounded by* $1 - \mu_2$ *for all* $s \geq 1$*.*

Then each stochastic matrix $Q(\ell)$*, has nonzero entries that can be lower bounded by a real* $\gamma > 0$*, arbitrarily small but finite (and independent of* ℓ*).*

Proof. The condition that is often required for convergence, that the nonzero entries of each $P(s)$ in the Markov chain are not smaller than a positive constant $\delta > 0$, can be expressed in terms of the entries of $R(s)$ in Eq. 6 of the main text: $[R(s)]_{ij} \geq \delta > 0$. Such a condition is always true by construction. In fact, from $V(s) = \left[\begin{array}{c|c} R(s) & 0 \end{array}\right] = (I - (I - \Theta(s))W(s))^{-1}\Theta$ we have that, since $(I - \Theta(s))W(s)$ is Schur stable, it can be expanded in Neumann series as

$$\sum_{r=0}^{\infty}((I - \Theta(s))W(s))^r = (I - (I - \Theta(s))W(s))^{-1}.$$

Since $W(s)$ is full and positive, every (i, j) entry of $(I - (I - \Theta(s))W(s))^{-1}$ is positive and obtained as a summation of nonnegative quantities each greater or equal to $[\sum_{r=0}^{\nu}((I - \Theta(s))W(s))^r]_{ij}$, where ν is the minimal power needed to reach each node from the stubborn nodes with $\theta_j(s) > 0$. For any choice of $W(s)$ and $\Theta(s)$, it is always possible to find $\delta' > 0$, which depends only on μ_1, μ_2, such that $[\sum_{r=0}^{\nu}((I - \Theta(s))W(s))^r]_{ij} \geq \delta'$, and hence also $[R(s)]_{ij} \geq \delta' > 0$, if $0 < \delta < \delta' \min_{\theta_i(s) \neq 0}(\theta_i(s))$. Since the intervals $[0, k_1], [k_1 + 1, k_2], \ldots$ have all finite length, also for $Q(\ell) = P(k_{\ell-1} + 1 : k_\ell)$ it is possible to find $\gamma > 0$ such that for all $\ell > 0$ each nonzero entry of $Q(\ell)$ is greater or equal than γ. ∎

Theorem 1 *Under the same assumptions of Proposition 2, and assuming in addition that* $\mathcal{R}(i_\ell, k_{\ell-1} : k_\ell) = \mathcal{V}$ *for all* $\ell = 1, 2, \ldots$*, then the backward Markov chain of Eq. 1 (and hence also Eq. 7 in the main text) achieves consensus as* ℓ *goes to infinity.*

Proof. The matrices $Q(\ell) = P(k_{\ell-1} + 1 : k_\ell)$, $\ell = 1, 2, \ldots$ in Eq. 1 are all stochastic. Therefore, we only need to prove that $Q(\ell) \ldots Q(2)Q(1)$ converges to a rank-1 matrix as ℓ goes to infinity. From Proposition 1, each $Q(\ell)$ has one positive column. According to the existing results (*16*), a sequence of products of stochastic matrices with a positive column converges to a rank-1 matrix if the positive entries of all the matrices can be lower bounded by a positive number. This is shown in Proposition 2. ∎

2.4 An alternative multidimensional model

As an alternative to the concatenated FJ model described in the main paper, we propose here another formulation, multidimensional in the opinion state representation. The concept is illustrated in Supplementary Fig. S11. Rather than concatenating the various FJ models of the constituted bodies in a purely sequential way, these models are run partly in parallel, partly according to a

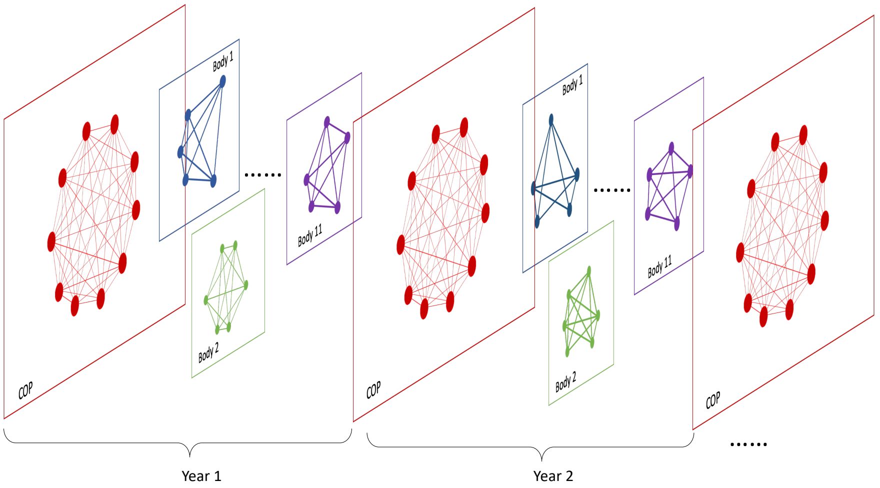

Fig. S11: Multidimensional FJ model for the achievement of consensus in the Paris Agreement. The opinion of a party is now represented by a vector. Each meeting of a constituted body updates one (or more) of the variables of the opinion vector, according to the topic that the body is assigned to deliberate on. The topics discussed in the meetings of the various constituted bodies are coupled according to a cross interdependence matrix, meaning that some bodies are influencing each other, while others are not. In the annual COP, all topics are discussed.

mutual cross-dependence pattern that expresses the interactions among the topics discussed by the various constituted bodies. A matrix of cross interdependencies can be used to express these interactions (*42, 43*). In this new model, for each party instead of a scalar opinion variable we consider an opinion vector, for instance (but not necessarily) one variable per constituted body. The rationale is that now the opinion of a party is broken down into multiple aspects (social, economical, financial, technological, political, etc.) denoted "topics", and each constituted body only discusses some (or perhaps just one) of these topics, meaning that only part of the state vector gets updated after the meeting of a constituted body. In the annual COP, all variables are instead updated because in a COP all topics are discussed. Specifically, we use $\mathcal{T} = \{1, \dots, r\}$ to denote the set of topics. The cross interdependency of these topics is described by a stochastic matrix, denoted $C = [c_{\ell k}]_{r \times r}$. Let $x^\ell(s,t) \in \mathbb{R}^n$ be the vector collecting all the opinions on topic ℓ at time t in meeting s, and $x_i^\ell(s,t)$ be the component of $x^\ell(s,t)$ relative to agent i. Define an indicator map $\mathrm{tp}(\cdot) : \mathbb{N} \mapsto 2^{\mathcal{T}}$ describing the sequence of discussed topics, i.e., $\mathrm{tp}(s) \subset \mathcal{T}$ represents the subset of topics discussed in meeting s. For $i \in \mathcal{V}$, $\ell \in \mathcal{T}$ and $s, t \geq 0$, the opinion dynamics can be described as follows:

$$
\begin{aligned}
x_i^\ell(s,t+1) &= \begin{cases} \theta_i(s) \sum_j w_{ij}(s) x_j^\ell(s,t) + (1-\theta_i(s)) x_i^\ell(s,0), & i \in \mathcal{M}(s) \text{ and } \ell \in \mathrm{tp}(s), \\ x_i^\ell(s,t), & i \notin \mathcal{M}(s) \text{ or } \ell \notin \mathrm{tp}(s), \end{cases} \\
x_i^\ell(s+1,0) &= \begin{cases} \sum_{k=1}^r c_{\ell k} x_i^k(s,\infty), & i \in \mathcal{M}(s) \text{ and } \ell \in \mathrm{tp}(s), \\ x_i^\ell(s,\infty), & i \notin \mathcal{M}(s) \text{ or } \ell \notin \mathrm{tp}(s). \end{cases}
\end{aligned} \tag{3}
$$

For subsequent use, given an $r \times r$ matrix $B = [b_{ij}]$ and two sets $\mathcal{A}_1, \mathcal{A}_2$, we define an $r \times r$ matrix $B_{\mathcal{A}_1:\mathcal{A}_2}$ as

$$[B_{\mathcal{A}_1:\mathcal{A}_2}]_{ij} = \begin{cases} b_{ij}, & \text{if } i \in \mathcal{A}_1 \text{ and } j \in \mathcal{A}_2, \\ 0, & \text{otherwise.} \end{cases} \tag{4}$$

Denoting $\mathbf{x}(s,t) = ((x^1(s,t))^\top, \dots, (x^r(s,t))^\top)^\top$, the opinion formation can be written in compact form as

$$\mathbf{x}(s+1,0) = H(s)\mathbf{x}(s,0), \tag{5}$$

with

$$H(s) = C_{\mathrm{tp}(s):\mathrm{tp}(s)} \otimes P_{\mathcal{M}(s):\mathcal{M}(s)}(s) + C_{\mathrm{tp}(s):\mathrm{tp}(s)^\complement} \otimes I_{\mathcal{M}(s):\mathcal{M}(s)} + I_{\mathrm{tp}(s):\mathcal{T}} \otimes I_{\mathcal{M}(s)^\complement:\mathcal{M}(s)^\complement}(s) + I_{\mathrm{tp}(s)^\complement:\mathcal{T}} \otimes I_n, \tag{6}$$

where $P(s)$ is defined in Eq. 6 in the main text and $\mathrm{tp}(s)^\complement$ (reps. $\mathcal{M}(s)^\complement$) represents the complement set of $\mathrm{tp}(s)$ in $\mathcal{T}$ (resp. $\mathcal{M}(s)$ in $\mathcal{V}$). Of the 4 terms of Eq. 6, the first two describe the evolution of the opinions in $\mathrm{tp}(s)$ of the agents participating in the s-th meeting. The second term in particular represents the influence of the self opinions about the non-discussed topics (i.e., $\mathrm{tp}(s)^\complement$) of the participants (see the third line of Eq. 3, the sum not only includes topics in $\mathrm{tp}(s)$ but also those in $\mathrm{tp}(s)^\complement$). The third term represents the evolution of the opinions in $\mathrm{tp}(s)^\complement$ of the participants, and the fourth the opinions of the agents absent in the s-th meeting.

Structure of the multidimensional opinion dynamics applied to the Paris Agreement data. For the sake of simplicity, from now on we associate a single topic with each constituted body. Since we are considering 11 bodies of the UNFCCC program, it is $\mathcal{T} = \{1, \dots, 11\}$. During the annual COP meeting, all parties are present and all topics are discussed. Therefore, the topic set of the k-th COP is $\mathrm{tp}^{\mathrm{COP}}(k) = \mathcal{T}$, and the stochastic matrix $P^{\mathrm{COP}}(k)$ has the same form as Eq. 11 in the main text, which means that $P^{\mathrm{COP}}(k)$ has at least one positive column.

There are two possible ways to intend consensus in the multidimensional model we are considering

A1: all parties converge to the same consensus value on all topics, i.e.,

$$\lim_{s\to\infty} x_i^\ell(s,t) = \lim_{s\to\infty} x_j^k(s,t) \quad \forall\, i,j = 1\dots,n, \quad \forall\, k,\ell \in \mathcal{T}.$$

A2: all parties reach an agreement on each topic but different topics correspond to different consensus values, i.e.,

$$\lim_{s\to\infty} x_i^\ell(s,t) = \lim_{s\to\infty} x_j^\ell(s,t) \quad \forall\, i,j = 1\dots,n,$$
$$\lim_{s\to\infty} x_i^\ell(s,t) \neq \lim_{s\to\infty} x_i^k(s,t) \quad k,\ell \in \mathcal{T}.$$

Concerning the case A1, there are two possibilities also for what concerns the choice of stochastic matrix to associate to the COP: the first possibility is to keep considering the same cross interdependence matrix C used for the constituted bodies, i.e.,

$$H^{\mathrm{COP}}(k_\ell) = C \otimes P^{\mathrm{COP}}(k_\ell) \tag{7}$$

where k_ℓ, $\ell = 1, 2, \ldots$, are the indexes of the COPs. The second possibility is instead to disregard C and treat the topics at a COP as independent:

$$H^{\mathrm{COP}}(k_\ell) = I \otimes P^{\mathrm{COP}}(k_\ell). \tag{8}$$

Let us consider Eq. 7 first. Since in a COP $\mathrm{tp}(s)^{\complement} = \emptyset$ and $\mathcal{M}(s)^{\complement} = \emptyset$, in Eq. 6 the last three terms are 0 and only the first term remains. We need to make the assumptions that the graph of C has a spanning tree and its diagonal entries are all positive (both are reasonable assumptions for the problem under investigation). More precisely, suppose C has a spanning tree rooted in ℓ' and the j'-th column of $P^{\mathrm{COP}}(s)$ is positive. Construct a trellis graph with each layer consisting of rn nodes, indexed by (ℓ, i) for $\ell \in [r], i \in [n]$. For simplicity, we focus on the trellis graph where the edges between layer 0 and layer 1 correspond to $H^{\mathrm{COP}}(s)$, and the edges between layer 1 and layer 2 correspond to $H(s+1) \ldots$ We have $\mathcal{R}((\ell', j'), 0:1) = \{(\ell, j) | c_{\ell\ell'} > 0, i \in \mathcal{V}\}$ (the symbol $\mathcal{R}(\cdot, \cdot)$ describes reachable sets, as defined in the paper). Consider layer 2. Observe that $\mathcal{R}((\ell', j'), 0:1) \subset \mathcal{R}((\ell', j'), 0:2)$, since for any $(\ell, j) \in \mathcal{R}((\ell', j'), 0:1)$, it holds

- if $\ell \in \mathrm{tp}(s+1)$, there is a path $(\ell', j') \to (\ell, \tilde{j}) \to (\ell, j)$, where $\tilde{j} \in \mathcal{V}$ is such that $[P(s+1)]_{j\tilde{j}} > 0$;
- if $\ell \notin \mathrm{tp}(s+1)$, there is a path $(\ell', j') \to (\ell, j) \to (\ell, j)$.

This process can be iterated. Therefore, we have $\{(\ell, \mathcal{V}) | c_{\ell'\ell} > 0\} \subset \mathcal{R}((\ell', j'), 0:k)$ for all $k > 0$. By the same analysis as above, we can show that if the $(s+k_1)$-th meeting is the first COP since s, it holds $\{(\ell, j) | [C^2]_{\ell\ell'} > 0, j \in \mathcal{V}\} \subset \mathcal{R}((\ell', j'), 0:k_1+1)$, and further $\{(\ell, j) | [C^2]_{\ell\ell'} > 0, j \in \mathcal{V}\} \subset \mathcal{R}((\ell', j'), 0:k)$ for all $k \geq k_1 + 1$. Iterating the process to any interval $[s, s+k]$ long enough to include the indexes of d COPs (here d is the diameter of the graph corresponding to C), the product of matrices $H(s+k) \ldots H(s+1)H(s)$ has a positive column. Since the interval between any two COPs has a bounded length, all the countries on all the topics will eventually reach agreement.

As a matter of fact, the assumption that C has a spanning tree can be weakened to C having a spanning forest. In fact, any C with a spanning forest can be written in block-diagonal form, with each block on the diagonal having a spanning tree. Then by the same analysis as above for all the diagonal blocks, we can show that the opinions of all countries on the topics corresponding to one block will reach consensus, while the opinions on topics in different blocks can be different (this would correspond to the case A2).

For the case A1, if we assume instead that during a COP the cross interactions between topics become irrelevant, then we have Eq. 8, for which the conclusion above still holds. For the sake of completeness, we give here a proof. Let the meeting index of the COPs be $k_1, k_2, \ldots$ We have the following observations for our problem:

1. there exists $K > 0$ such that $k_{\ell+1} - k_\ell < K$ for all $\ell = 1, 2, \ldots$
2. it holds that $\mathrm{tp}(k_\ell + 1) \cup \cdots \cup \mathrm{tp}(k_{\ell+1} - 1) = \mathcal{T}$ for all $\ell = 1, 2, \ldots$

Again consider the trellis graph constructed as mentioned above, and let the first meeting be a COP, i.e., $k_1 = 1$. Assume C has a spanning tree rooted in ℓ', and its diagonal entries are all positive. Let the j'-th column of $P^{\mathrm{COP}}(1)$ be positive. Similar to the statements above, we

obtain $\{(\ell', j) | j \in \mathcal{V}\} \subset \mathcal{R}((\ell', j'), 0 : k)$. As $\text{tp}(2) \cup \cdots \cup \text{tp}(k_2 - 1) = \mathcal{T}$, for any ℓ with $c_{\ell\ell'} > 0$, there exists $k < k_2$ such that $\ell \in \text{tp}(k)$. Therefore, for all $j \in \mathcal{V}$, there is a path $(\ell', j') \to \cdots \to (\ell', \tilde{j}) \to (\ell, j)$, where $\tilde{j}$ is such that $[P(k)]_{j\tilde{j}} > 0$, and the nodes on the path from (ℓ', j') to ℓ', j' are all indexed in the form $(\ell', \bar{j}), j \in \mathcal{V}$, with any two consecutive nodes $(\ell', \bar{j}_1), (\ell', \bar{j}_2)$ in layer $s', s' + 1$ being such that $[P(s' + 1)]_{\bar{j}_2\bar{j}_1} > 0$. Also by the same reasoning as above, we obtain $\{(\ell, j) | j \in \mathcal{V}\} \subset \mathcal{R}((\ell', j'), 0 : k')$ for all $k' \geq k$. As ℓ is arbitrarily taken such that $c_{\ell\ell'} > 0$, we have $\{(\ell, j) | c_{\ell\ell'} > 0, j \in \mathcal{V}\} \subset \mathcal{R}((\ell', j'), 0 : k'))$ for all $k' \geq k_2 - 1$. By the same argument, we can show that $\{(\ell, j) | [C^2]_{\ell\ell'} > 0, j \in \mathcal{V}\} \subset \mathcal{R}((\ell', j'), 0 : k'))$ for all $k' \geq k_3 - 1$. Repeating the analysis, we have $\mathcal{R}((\ell', j'), 0 : k_{d+1} - 1) = \{(\ell, j) | \ell \in \mathcal{T}, j \in \mathcal{V}\}$, where d is the diameter of the graph corresponding to C. This means that the matrix product $H(k_{d+1} - 1) \cdots H^{\text{COP}}(1)$ has a positive column, i.e., the column indexed by (ℓ', j'). This property also holds for $H(k_{2d+1} - 1) \cdots H(k_{d+1}), H(k_{3d+1} - 1) \cdots H(k_{2d+1}), \ldots$ By the first observation above, we know that $k_{md+1} - k(m-1)d + 1 < dK$. Therefore, the matrix product $H(k) \cdots H(1)$ converges to some rank-1 matrix as k approaches infinity. We then know that all the opinions on all topics will converge to the same value.

Concerning the case A2, a different consensus value on each topic is achieved with Eq. 8 because $P^{\text{COP}}(k)$ with a positive column is enough to have the reachability condition satisfied periodically. The analysis can then be carried out along similar lines.

Practical aspects As for the model discussed in the paper, a convergence always requires an infinite time horizon. Thus for our dataset (with its finite time horizon) we can expect at most "practical consensus".

The cross interdependency matrix C used in the multidimensional model describes the mutual interactions and dependencies among the topics discussed in the various meetings. If we identify the topics with the constituted bodies, then C is a 11×11 row stochastic matrix. Since an entry on the diagonal represents how much an opinion about a specific topic is "anchored" to that topic (i.e., how much a constituted body can act autonomously on the topic it is created for), we assume that its value is significantly greater than those outside the diagonal. In particular, we suppose $c_{ii} = 0.75$ for each $i = 1, \ldots, 11$. The entries outside the diagonal represent the cross-influences between the constituted bodies. The following considerations have been made to identify their values:

- AC, CGE, LEG, SCF and TEC are committees under the Convention, while the rest are under the Kyoto Protocol;
- CTCN membership includes a representative from AC, one from SCF and one from TEC;
- the activities of AFB, CTCN, CGE, CDM-EB and LEG concern developing countries;
- CC-E and CC-F form the Compliance Committee of the Kyoto Protocol;
- both AC and CC-F provide guidelines and recommendations;
- both AFB and SCF deal with financial aspects.

Based on these considerations, we have decided to use the following cross interdependency matrix:

$$
\begin{array}{cc}
 & \begin{array}{ccccccccccc} \text{AC} & \text{AFB} & \text{CTCN} & \text{CC-E} & \text{CC-F} & \text{CGE} & \text{CDM-EB} & \text{JISC} & \text{LEG} & \text{SCF} & \text{TEC} \end{array} \\
C = & \begin{bmatrix}
0.75 & 0 & 0.06 & 0 & 0.03 & 0.02 & 0 & 0 & 0.02 & 0.06 & 0.06 \\
0 & 0.75 & 0.03 & 0.03 & 0.03 & 0 & 0.08 & 0.06 & 0 & 0.02 & 0 \\
0.05 & 0.02 & 0.75 & 0 & 0 & 0.02 & 0.02 & 0 & 0.02 & 0.04 & 0.08 \\
0 & 0.03 & 0 & 0.75 & 0.16 & 0 & 0.03 & 0.03 & 0 & 0 & 0 \\
0.03 & 0.02 & 0 & 0.16 & 0.75 & 0 & 0.02 & 0.02 & 0 & 0 & 0 \\
0.03 & 0 & 0.08 & 0 & 0 & 0.75 & 0 & 0 & 0.08 & 0.03 & 0.03 \\
0 & 0.10 & 0.04 & 0.03 & 0.03 & 0 & 0.75 & 0.05 & 0 & 0 & 0 \\
0 & 0.03 & 0 & 0.08 & 0.07 & 0 & 0.07 & 0.75 & 0 & 0 & 0 \\
0.03 & 0 & 0.08 & 0 & 0 & 0.08 & 0 & 0 & 0.75 & 0.02 & 0.04 \\
0.06 & 0.03 & 0.06 & 0 & 0 & 0.02 & 0 & 0 & 0.02 & 0.75 & 0.06 \\
0.06 & 0 & 0.09 & 0 & 0 & 0.02 & 0 & 0 & 0.02 & 0.06 & 0.75
\end{bmatrix}
\begin{array}{l} \text{AC} \\ \text{AFB} \\ \text{CTCN} \\ \text{CC-E} \\ \text{CC-F} \\ \text{CGE} \\ \text{CDM-EB} \\ \text{JISC} \\ \text{LEG} \\ \text{SCF} \\ \text{TEC} \end{array}
\end{array}
$$

REFERENCES AND NOTES

1. M. H. DeGroot, Reaching a consensus. *J. Am. Stat. Assoc.* **69**, 118–121 (1974).

2. P. Jia, A. Mirtabatabaei, N. E. Friedkin, F. Bullo, Opinion dynamics and the evolution of social power in influence networks. *SIAM Rev Soc Ind Appl Math* **57**, 367–397 (2015).

3. A. Mirtabatabaei, P. Jia, N. E. Friedkin, F. Bullo, On the reflected appraisals dynamics of influence networks with stubborn agents, in *Proceedings of the 2014 American Control Conference* (IEEE, 2014), 3978–3983.

4. M. Ye, J. Liu, B. D. Anderson, C. Yu, T. Başar, Evolution of social power in social networks with dynamic topology. *IEEE Trans. Automat. Contr.* **63**, 3793–3808 (2018).

5. Y. Tian, P. Jia, A. Mirtabatabaei, L. Wang, N. E. Friedkin, F. Bullo, Social power evolution in influence networks with stubborn individuals. arXiv:1901.08727 (2019).

6. Y. Tian, L. Wang, Opinion dynamics in social networks with stubborn agents: An issue-based perspective. *Automatica* **96**, 213–223 (2018).

7. N. E. Friedkin, E. C. Johnsen, Social influence and opinions, *J. Math. Sociol.* **15**, 193–206 (1990).

8. N. Friedkin, E. Johnsen, *Social Influence Network Theory: A Sociological Examination of Small Group Dynamics* (Structural Analysis in the Social Science, Cambridge Univ. Press, 2011).

9. J. Wolfowitz, Products of indecomposable, aperiodic, stochastic matrices. *Proc. Am. Math. Soc.* **14**, 733–737 (1963).

10. L. Moreau, Stability of multiagent systems with time-dependent communication links. *IEEE Trans. Automat. Contr.* **50**, 169–182 (2005).

11. V. D. Blondel, J. M. Hendrickx, A. Olshevsky, J. N. Tsitsiklis, Convergence in multiagent coordination, consensus, and flocking, in *Proceedings of the 44th IEEE Conference on Decision and Control* (2005), pp. 2996–3000.

12. M. Cao, A. S. Morse, B. D. O. Anderson, Reaching a consensus in a dynamically changing environment: A graphical approach. *SIAM J. Contr. Optim.* **47**, 575–600 (2008).

13. D. Angeli, P.-A. Bliman, Convergence speed of unsteady distributed consensus: Decay estimate along the settling spanning-trees. *SIAM J. Contr. Optim.* **48**, 1–32 (2009).

14. J. M. Hendrickx, J. N. Tsitsiklis, Convergence of type-symmetric and cut-balanced consensus seeking systems. *IEEE Trans. Automat. Contr.* **58**, 214–218 (2013).

15. B. Touri, A. Nedić, Product of random stochastic matrices. *IEEE Trans. Automat. Contr.* **59**, 437–448 (2013).

16. B. Touri, A. Nedić, On backward product of stochastic matrices. *Automatica* **48**, 1477–1488 (2012).

17. D. Klein, M. Carazo, M. Doelle, J. Bulmer, A. Higham, *The Paris Agreement on Climate Change: Analysis and Commentary* (Oxford Scholarly Authorities on International Law, Oxford Univ. Press, 2017).

18. L. Ø. Blaxekjær, T. D. Nielsen, Mapping the narrative positions of new political groups under the UNFCCC. *Clim. Policy* **15**, 751–766 (2015).

19. G. Edwards, I. C. Adarve, M. C. Bustos, J. T. Roberts, Small group, big impact: how AILAC helped shape the Paris Agreement. *Clim. Policy* **17**, 71–85 (2017).

20. K. Michaelowa, A. Michaelowa, India as an emerging power in international climate negotiations. *Clim. Policy* **12**, 575–590 (2012).

21. K. Bäckstrand, O. Elgström, The EU’s role in climate change negotiations: From leader to ‘leadiator’. *J. Eur. Publ. Policy* **20**, 1369–1386 (2013).

22. P. Castro, National interests and coalition positions on climate change: A text-based analysis. *Int. Polit. Sci. Rev.* **42**, 95–113 (2021).

23. P. Tobin, N. M. Schmidt, J. Tosun, C. Burns, Mapping states' Paris climate pledges: Analysing targets and groups at COP 21. *Glob. Environ. Chang.* **48**, 11–21 (2018).

24. V. Costantini, G. Sforna, M. Zoli, Interpreting bargaining strategies of developing countries in climate negotiations. A quantitative approach. *Ecol. Econ.* **121**, 128–139 (2016).

25. K. Lessmann, U. Kornek, V. Bosetti, R. Dellink, J. Emmerling, J. Eyckmans, M. Nagashima, H.P. Weikard, Z. Yang, The stability and effectiveness of climate coalitions. *Environ. Resource Econ.* **62**, 811–836 (2015).

26. N. E. Friedkin, E. C. Johnsen, Influence networks and opinion change. *Adv. Group Processes* **16**, 1–29 (1999).

27. J. Depledge, *The Organization of Global Negotiations: Constructing the Climate Change Regime* (Taylor & Francis, 2013).

28. N. E. Friedkin, The problem of social control and coordination of complex systems in sociology: A look at the community cleavage problem. *IEEE Contr. Syst. Mag.* **35**, 40–51 (2015).

29. S. Chatterjee, E. Seneta, Towards consensus: Some convergence theorems on repeated averaging. *J. Appl. Probab.* **14**, 89–97 (1977).

30. N. E. Friedkin, F. Bullo, How truth wins in opinion dynamics along issue sequences. *Proc. Natl. Acad. Sci.* **114**, 11380–11385 (2017).

31. R. Perloff, *The Dynamics of Persuasion: Communication and Attitudes in the Twenty-First Century* (Routledge Communication Series, Taylor & Francis, 2016).

32. J. Folger, M. Poole, R. Stutman, *Working Through Conflict: Strategies for Relationships, Groups, and Organizations* (Taylor & Francis, 2021).

33. D. A. Infante, C. J. Wigley III, Verbal aggressiveness: An interpersonal model and measure. *Commun. Monogr.* **53**, 61–69 (1986).

34. M. Egidi, A. Narduzzo, The emergence of path-dependent behaviors in cooperative contexts. *Int. J. Ind. Organ.* **15**, 677–709 (1997).

35. R. MacKay, S. Masrani, P. McKiernan, Strategy options and cognitive freezing: The case of the Dundee jute industry in Scotland. *Futures* **38**, 925–941 (2006).

36. R. Wurzel, J. Connelly, *The European Union as a Leader in International Climate Change Politics* (Routledge/UACES Contemporary European Studies, Taylor & Francis, 2010).

37. M. del Pilar Bueno, G. Pascual, International climate framework in the making: the role of the BASIC countries in the negotiations towards the Paris agreement. *Janus.net:e-journal of International Relations* **7**, 122–140 (2016).

38. J. Kuyper, H. Schroeder, B.-O. Linner, The Evolution of the UNFCCC. *Annu. Rev. Env. Resour.* **43**, 343–368 (2018).

39. S. Afionis, I. Chatzopoulos, Russia's role in UNFCCC negotiations since the exit of the United States in 2001, *Int. Environ. Agreements* **10**, 45–63 (2010).

40. F. Bullo, *Lectures on Network Systems* (Kindle Direct Publishing, 2021).

41. F. Fagnani, P. Frasca, *Introduction to Averaging Dynamics over Networks* (Springer, 2018).

42. S. E. Parsegov, A. V. Proskurnikov, R. Tempo, N. E. Friedkin, Novel multidimensional models of opinion dynamics in social networks. *IEEE Trans. Automat. Contr.* **62**, 2270–2285 (2016).

43. M. Ye, M. H. Trinh, Y.-H. Lim, B. D. Anderson, H.-S. Ahn, Continuous-time opinion dynamics on multiple interdependent topics. *Automatica* **115**, 108884 (2020).
